# Advancing drug development for systemic sclerosis by prioritizing findings from human genetic association studies

**DOI:** 10.1093/rheumatology/keag084

**Published:** 2026-02-13

**Authors:** Michael Hughes, Zsuzsanna H McMahan, Shervin Assassai, Christopher P Denton, Rui Providencia

**Affiliations:** Centre for Musculoskeletal Research, Division of Musculoskeletal and Dermatological Science, School of Biological Sciences, Faculty of Biological Medicine and Health, Manchester Academic Health Science Centre, The University of Manchester, Manchester, UK; Department of Rheumatology, Northern Care Alliance NHS Foundation Trust, Salford Care Organisation, Salford, UK; NIHR Manchester Biomedical Research Centre, Manchester University NHS Foundation Trust, Manchester, UK; Department of Medicine, Division of Rheumatology, UTHealth Houston, Houston, TX, USA; Department of Medicine, Division of Rheumatology, UTHealth Houston, Houston, TX, USA; Division of Medicine, University College London, London, UK; Institute of Health Informatics Research, University College London, London, UK; Barts Heart Centre, St Bartholomew’s Hospital, London, UK

**Keywords:** systemic sclerosis, scleroderma, drug repurposing, clinical trials

## Abstract

**Objectives:**

SSc is a rare rheumatological disease associated with significant morbidity and mortality. Despite significant recent international clinical trial activity, the yield of approved compounds has been disappointingly low. Our aim was to identify and prioritize potential ‘druggable’ targets with insights from human genetics, by integrating the available evidence with publicly available bioinformatics sources relevant for SSc drug development.

**Methods:**

Genetic variants for SSc were identified through a search of the GWAS Catalog, and the associated-mapped genes were cross-referenced with the OpenTargets platform for drug interactions. Confirmation/validation was demonstrated through structured literature searches and review of the evidence on MEDLINE and ClinialTrials.gov for each individual drug and its association with SSc.

**Results:**

We identified 89 unique drugs, none of which is included in existing SSc guidelines/recommendations. Amlitelimab (anti-OX40L mAb) is currently being explored in CONQUEST (Platform Clinical Study for Conquering Scleroderma), a multicentre randomized controlled platform trial for SSc interstitial lung disease. Key groupings of drug therapies were (i) female sex hormones and function, (ii) neurotransmitter-targeting therapies, and (iii) inflammatory–fibrotic pathways. The Janus kinase (JAK)/STAT pathway is an attractive therapeutic target in SSc, targeting known pathobiology.

**Conclusion:**

Our systematic approach, combining evidence from different bioinformatics platforms, has identified drug opportunities for repurposing/druggable targets for SSc. A novel and unexpected finding was the identification of multiple neurotransmitter-targeting drug therapies, particularly relevant to SSc-related RP and gastrointestinal involvement. Future studies of these candidates for SSc drug repurposing, many of which are widely available and often inexpensive, are indicated.

Rheumatology key messages

- We have identified many potential opportunities for drug repurposing with druggable targets for SSc.
- Key groupings of targets were: female sex hormones and function, neurotransmitter-targeting therapies and inflammatory–fibrotic pathways.
- Future studies of candidates for SSc drug repurposing, many of which are available/inexpensive, are indicated.

## Introduction

SSc is a complex rheumatological condition with incompletely understood pathogenesis spanning autoimmunity, vasculopathy and aberrant tissue fibrosis [1, 2]. SSc occurs in genetically susceptible individuals, and other (e.g. environmental and/or epigenetic) factors likely have important roles in disease pathobiology [3]. Although progress has been made in contemporary management, including with the approval of drug therapies for some aspects [4, 5], the disease is still responsible for significant morbidity and mortality [6, 7]. Notable improvements in SSc pharmacological management include nintedanib and tocilizumab for the treatment of interstitial lung disease (ILD) [8, 9], and bosentan for refractory digital ulcers [10]. Autologous stem-cell transplantation represents a highly powerful form of systemic immunosuppression and can be transformative in highly selected individuals. Newer cellular strategies, including chimeric antigen receptor T-cell therapies, are emerging. However, such intensive approaches are not risk-free and are reserved for those with the highest risk of disease progression and poorer outcomes [4].

Both the rarity of SSc and disease heterogeneity are major challenges to successful drug development and expensive clinical trial programs [11, 12]. Recognizing a fundamental global research challenge, it has been estimated that <5% of drug development programs ultimately yield licensed drugs [13]. In SSc, there has been an understandable focus on drug development for patients with the early diffuse cutaneous subset of the disease, which has a high risk of poor outcomes, including through organ-based involvement [14, 15]. However, recent efforts have highlighted the significant burden of the (more common) limited cutaneous subset of the disease, and similar justification for newer therapeutic options [16, 17]. Another consideration is that with improved outcomes seen in many contemporary patients, later-stage survivors of the disease should not be forgotten by the successes of modern advances in SSc management [7, 18]. Furthermore, much of the lived non-lethal morbidity of the disease in SSc is currently difficult to treat (and little understood), reinforcing the need to explore novel targeted therapies informed by likely important pathogenetic mechanisms.

There have been notable recent developments and maturation of (i) relatively low-cost nucleic acid sequencing platforms, and (ii) advanced statistical tools to generate inferences from genomic data. As such, a wealth of knowledge has been accumulated concerning the genetic associations of a range of common human diseases. This includes many genetic studies, including candidate gene analysis and genome-wide association studies (GWAS), which have identified associated genetic variants (mainly localized in noncoding regions) in the expression of quantitative traits, yet influencing gene expression. In SSc, a wide range of genes (both HLA and non-HLA regions) have been implicated in disease susceptibility, including observed clinical phenotype [19, 20]. Furthermore, HLA class II genes are reported to be associated with SSc-related autoantibodies [19], rather than the disease itself. Novel associations with HLA class I genes have also been reported in SSc [20]. However, to date, these rich insights into the genetic landscape of SSc have not directly translated into new targeted drug treatments for the disease. In parallel, the relatively recent availability of large open-source libraries of bioactive molecules with drug-like properties, including protein sequencing and functional information, provides insight into potentially ‘druggable’ proteins [21].

By adopting an integrated approach, the predicted pharmacological effects of targeting implicated gene variants can be modelled to existing, well-characterized, drug therapies [13]. This approach provides an effective mechanism by which screen a large number of candidate drug therapies against genetically identified targets [22]. A major advantage of this approach is that these drug therapies are often at later stages of drug development processes for other indications, and thereby more likely to progress (and with efficiencies to be saved) through clinical trials for other indications [22]. A previous study (reported over a decade ago) highlighted potential drug repositioning among related systemic seropositive rheumatic diseases, using identified protein targets of functional interest using publicly available databases [23].

Against this background, our aims were to identify in SSc: (i) potential targets for drug development where no active compounds are currently available, and (ii) possibilities for drug repurposing from their originally approved indication.

## Methods

In summary, we have built on the proven success of a similar approach [21], which investigated drug development for atrial fibrillation by prioritizing findings from human genetic association studies [24]. Our full study protocol is available elsewhere (protocols.io) [25]. Ethical review was not required as ours was a systematic examination of freely, publicly available data and did not include use of any personal identifiable information. Data were analysed using embedded software tools for data collation and interpretation. Patient consent was not required for this evidence synthesis as it involved the secondary analysis of publicly available, de-identified summary-level data. The original studies were conducted under the ethical oversight of their respective Institutional Review Boards and obtained broad consent for data sharing in accordance with established genomic data sharing policies.

### Selection of genetic association study data

We searched for genetic variants concerning SSc using the terms ‘scleroderma’ or ‘systemic scleroderma’ [26]. The search was conducted from inception to late December 2024 and then completely rerun on 3 August 2025 and 25 November 2025. GWAS, as well as other studies (e.g. those employing whole-genome sequencing or copy number variant association analyses) were also considered to identify variants associated with SSc. We also considered studies concerning SSc-related traits (e.g. internal organ complications), with the rationale being that these might identify further genes related to the disease itself and/or replicate those found in SSc association analyses. Information on genetic variants and their corresponding genes was extracted from the GWAS Catalog, which combines author-provided data with standardized processing through a pipeline that maps all genetic variants to the latest human genome build and Ensembl database to consistently identify the nearest genes (or genes within a certain distance).

We report associations that meet the standard genome-wide significance threshold, *P* < 5 × 10^−8^. Other associations from the GWAS Catalog were moved to the supplementary data file and excluded from the manuscript.

### Cross-referencing with bioinformatics database

Analogous to our previous study [24], we analysed the associated-mapped genes using the OpenTargets platform, which systematically integrates publicly available datasets to allow identification and prioritization of potential therapeutic drug targets [22].

Upon entering a gene of interest (e.g. *ESR1*), the platform interface provides comprehensive information, including its Ensembl and UniProt IDs, associated diseases, drug tractability, tissue and subcellular expression data, and Gene Ontology and pathway annotations. Supplementary Fig. S1 provides a more detailed tutorial.

### Literature review for identified drugs

For each of the identified drugs we performed dedicated searches of the PubMed^®^ database and ClinicalTrials.gov from inception until 3 and 4 August 2025. Our intention was to identify existing clinical data in humans related to drug efficacy and/or safety of relevance to SSc. The search expression/strategy used for our PubMed searches was ‘drug name’ AND ((‘systemic sclerosis’) OR (‘scleroderma’)). We considered relevant systematic reviews, randomized clinical trials (RCTs), and controlled studies performed in humans, which compared the drug *vs* placebo/active control, for the purpose of constructing our evidence synthesis tables. Concerning, ClinicalTrials.gov, we searched for ‘scleroderma’, ‘systemic scleroderma’ or ‘systemic sclerosis’ in the ‘Condition or Disease’ field, and the specific drug name was entered in the ‘Intervention/treatment’ field. In general, we sought to ascertain broad data pertaining to SSc, including clinical phenotype and internal organ complications.

## Results

**Figure 1:**
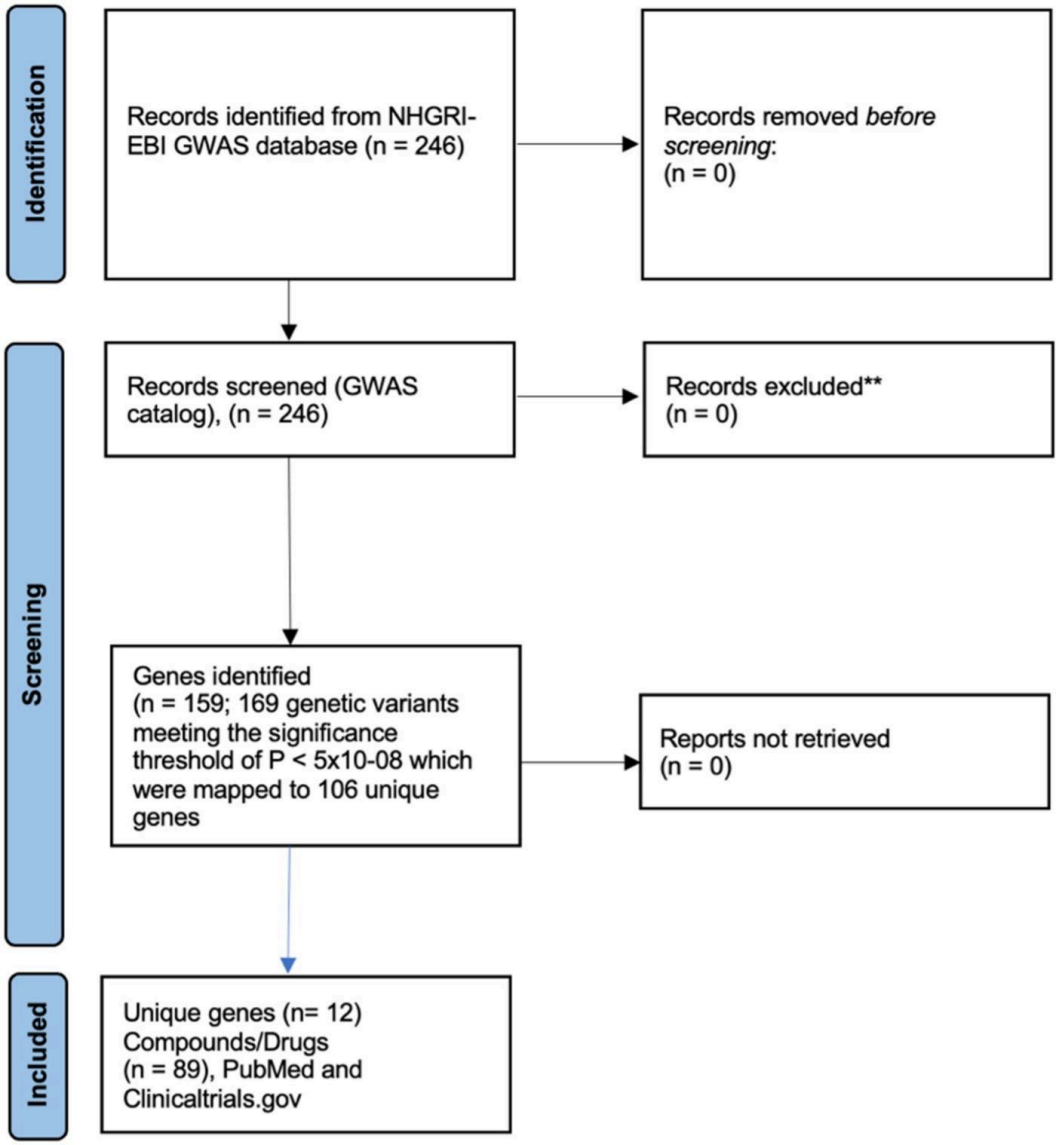
Study workflow and gene and drug selection process [49]; 89 unique drug compounds were identified, including variations (e.g. conjugated salts)

An overview of the study workflow is presented in Fig. 1. The search of the GWAS Catalog produced 246 records concerning 169 genetic variants meeting the significance threshold of *P* < 5 × 10^−8^ which were mapped to 106 unique genes considered of relevance to our study (Supplementary Table S1). When a genetic variant was mapped to two genes, such as rs4317244-G being mapped to both *ANKRD37* and *UFSP2*, each of the associated genes was independently assessed within the Open Targets Platform. Of this list, we identified 12 unique genes (*BLK*, *DRD4*, *ESR1*, *FCGR2B*, *GLS*, *HLA*-*DRB1*, *HLA*-DRB5, *NFKB1*, *PTPN11*, *STAT3*, *TNFSF4* and *TYK2*) and 89 unique drug (including variations, e.g. conjugate) compounds (Table 1) associated with the targets. Clinical evidence on the available drugs targeting identified hits that may constitute repurposing opportunities in SSc is presented in Table 1.

**Table 1:** Drugs targets, and summary of current clinical evidence, for potential repurposing in SSc.

Drug (phase)	Drug type	Target	Clinical indication	Clinical evidence regarding drug SSc outcomes	Maximum level of evidence	**Entries on PubMed** ^a^	**Entries on ClinicalTrials.gov** ^a^
Female sex hormones and function							
Acolbifene	SERM	ESR1			No controlled studies	0	0
Afimoxifene	SERM	ESR1			No controlled studies	1	0
Amcenestrant	SERD	ESR			No controlled studies	0	0
Arzoxifene	SERM	ESR1			No controlled studies	0	0
Bazedoxifene (including acetate)	SERM	ESR1			No controlled studies	1	0
Brilanestrant	SERD	ESR			No controlled studies	0	0
Camizestrant	SERD	ESR1			No controlled studies	0	0
Clomiphene (including citrate)	SERM	ESR1			No controlled studies	0	0
Diethylstilbestrol	Synthetic nonsteroidal oestrogen	ESR1			No controlled studies	3	0
Droperidol	Dopamine D2 receptor antagonist	DRD4			No controlled studies	2	0
Elacestrant	SERD	ESR1			No controlled studies	0	0
Enclomiphene	SERM	ESR1			No controlled studies	0	0
Estetrol	Estetrol (E4)—a natural oestrogenic steroid normally produced by human fetal liver	ESR1			No controlled studies	0	0
Estradiol (including acetate and valerate)	Estradiol (E1)—female sex hormone	ESR1			No controlled studies	43	2
Estriol	Estriol (E3)—female sex hormone	ESR1		Two female patients with generalized, progressive SSc were treated with estriol for 10 months. Reported improvement in skin disease and RP, no improvement in internal involvement. Histopathological—reduction of homogenization of collagen bundles in the dermis of the affected forearms [48]	No controlled studies	2	0
Estrogens (including esterified and synthetic conjugated)	Female sex hormone	ESR1			No controlled studies	155	1
Estrone	Estrone (E1)^b^—female sex hormone	ESR1			No controlled studies	3	0
Estropipate	Semi-synthetic oestrogen	ESR1			No controlled studies	0	0
Ethinyl estradiol	Synthetic oestrogen	ESR1			No controlled studies	10	1
Fispemifene	SERM	ESR1			No controlled studies	0	0
Fulvestrant	Oestrogen receptor antagonist	ESR1			No controlled studies	2	0
Giredestrant	SERD	ESR1			No controlled studies	0	0
GTX-758	Selective oestrogen receptor alpha agonist	ESR1			No controlled studies	0	0
Lasofoxifene (including tartarate)	SERM	ESR1			No controlled studies	0	0
Ospemifene	SERM	ESR1			No controlled studies	0	0
Pimozide	Dopamine receptor antagonist	DRD4			No controlled studies	0	0
Polyestradiol phosphate	Oestrogen ester	ESR1			No controlled studies	0	0
SR16234 (TAS-108)	SERM	ESR1			No controlled studies	0	0
Tamoxifen	SERM	ESR1			No controlled studies	28	1
Toremifene	SERM	ESR1			No controlled studies	0	0
Inflammatory–fibrotic pathways							
AZD-1236	MMP9 and MMP12 inhibitor	MMP9			No controlled studies	0	0
Amlitelimab (2b)	Fully human, non-depleting, anti-OX40L monoclonal antibody	TNFSF4	SSc-ILD [27]		Randomized controlled study—recruiting^a^	1	1
Apolizumab	Humanized IgG1 monoclonal anti-HLA-DR β-chain antibody targets the antigen	HLA-DRB1/HLA-DRB5			No controlled studies	0	0
Brepocitinib	Dual inhibitor of TYK2 and JAK1	TYK2			No controlled studies	0	0
Batoprotafib	Protein tyrosine phosphatase non-receptor type 11 (SHP2) inhibitor	PTPN11			No controlled studies	0	0
Cerdulatinib	SYK/JAKi	TYK			No controlled studies	0	0
Cravacitinib	Oral, selective, allosteric tyrosine kinase 2 (TYK2) inhibitor	TYK2			No controlled studies	0	0
CTS-1027	MMP inhibitor	MMP9			No controlled studies	0	0
Danvatirsen	Generation 2.5 antisense oligonucleotide designed to downregulate the expression of human *STAT3* mRNA	STAT3			No controlled studies	0	0
Dasatinib (2)	Second-generation tyrosine kinase inhibitor	BLK	SSc-ILD	Open label (biomarker) study for SSc-ILD. 100 mg dasatinib once daily orally for up to 2 years (primary endpoint at 6 months). 65% of patients showed no progression of lung fibrosis, and 39% showed no progression of total ILD [47]	No controlled studies	16	1
Delgocitinib	JAKi	TYK2			No controlled studies	1	0
Deucravacitinib	Tyrosine kinase 2 (TYK2) inhibitor	TYK2			No controlled studies	3	0
Edasalonexent	NF-κB inhibitor	NFKB1			No controlled studies	0	0
ENMD-981693	Orally active Kinase Inhibitor	BLK			No controlled studies	0	0
Filgotinib (including maleate)	JAKi	TYK2			No controlled studies	3	0
Gusacitinib	Dual inhibitor of JAKs and spleen tyrosine kinase (SYK)	TYK2			No controlled studies	0	0
Ilorasertib	Dual Aurora/VEGF receptor kinase inhibitor	BLK			No controlled studies	0	0
Izencitinib	Pan-JAKi	TYK2			No controlled studies	0	0
LYM-1	LYM-1 is a murine IgG2a mAb (CART)	LYM-1			No controlled studies	0	0
Marmiastat	Oral MMP inhibitor	MMP9			No controlled studies	1	0
Nezulcitinib	Pan-JAKi	TYK2			No controlled studies	0	0
Obexelimab	Bifunctional, non-cytolytic, humanized monoclonal antibody that binds CD19 and Fc gamma receptor IIb to inhibit B cells, plasmablasts, and CD19-expressing plasma cells	FCGR2B			No controlled studies	0	0
Ocriplasmin	Proteolytic activity against protein components of the vitreous body and the vitreoretinal interface	LAMC2			No controlled studies	0	0
Oxelumab	Human monoclonal antibody against the OX40 ligand (OX40L)	TNFSF4			No controlled studies	0	0
Peficitinib	JAKi	TYK2			No controlled studies	2	0
Plovamer acetate	Immunomodulator; mechanism not fully elucidated: MHC blocker and T-cell receptor antagonist	HLA-DRB1			No controlled studies	278	0
Rebimastat	Broad-spectrum MMP	MMP9			No controlled studies	0	0
Ropsacitinib	Selective TYK2 inhibitor	TYK2					
Telaglenastat	Glutaminase inhibitor	GLS			No controlled studies	0	0
Tofacitinib (including citrate)	JAK1	TYK2	SSc	52-week pilot study, 33 patients received 5 mg TOF twice daily + 33 received 10 MTX 10 mg/weekly (randomly allocated). Significant reduction in mRSS: TOF −11.27 ± 3.89 compared with MTX (−2.27 ± 2.32) units. Reduction in US skin thickness and MSK involvement. 75% healing of DUs and no recurrence in TOF treated patients [28]	Randomized controlled study	43	3
Upadacitinib	JAKi	TYK2			No controlled studies	4	1
TG100-801	Dual inhibitor of both VEGFr2 and the Src family	BLK			No controlled studies	0	0
XL-228	Multitargeted protein kinase inhibitor	BLK			No controlled studies	0	0
Neurotransmitter-targeting therapies							
Andecaliximab	mAb inhibiting MMP 9	MMP9			No controlled studies	0	0
Apomorphine	Non-selective dopamine agonist	DRD4			No controlled studies	1	0
Bromocriptine	Dopamine D2 agonist	DRD4			No controlled studies	9	0
Chlorpromazine	Antipsychotic (first-generation), weak presynaptic inhibitor of dopamine reuptake	DRD4			No controlled studies	8	0
Clothiapine	Atypical antipsychotic of the dibenzothiazepine chemical class, targets multiple neurotransmitters	DRD4			No controlled studies	0	0
Ergoloid (including mesylates)	Ergot alkyloid; dual action of partial agonism/antagonism of adrenergic, dopaminergic and serotonergic receptors	DRD4			No controlled studies	1	0
Haloperidol (including lactate)	Primarily a dopaminergic D2-antagonist	DRD4			No controlled studies	2	0
Levomepromazine	Phenothiazine antipsychotic; including antagonism of dopamine, serotonin, histamine, alpha-adrenergic and muscarinic receptors	DRD4			No controlled studies	0	0
Loxapine (including succinate)	Dopamine D2 and serotonin 5-HT2A receptor antagonist	DRD4			No controlled studies	0	0
Olanzapine (including pamoate)	Dopamine and serotonin type 2 (5HT2) antagonist	DRD4			No controlled studies	1	0
Pergolide	Ergoline-based dopamine receptor agonist	DRD4			No controlled studies	0	0
Pramipexole	Dopamine receptor agonist	DRD4			No controlled studies	1	0
Promazine	Dual antagonism at dopamine and serotonin type 2 receptors	DRD4			No controlled studies	0	0
Ropinirole	Non-ergoline dopamine agonist	DRD4			No controlled studies	0	0
Rotigotine	Nonselective agonist of dopamine receptors	DRD4			No controlled studies	0	0
Sarizotan	Selective 5-HT1A receptor agonist and D2 receptor antagonist	DRD4			No controlled studies	0	0
Thioridazine	Phenothiazine antipsychotic, dopamine antagonist	DRD4			No controlled studies	0	0
Trifluoperazine	Dopamine D1/D2 receptor antagonist	DRD4			No controlled studies	0	0

a Platform ‘CONQUEST’ SSc-ILD RCT [47]—currently recruiting.

b Estrone mainly serves as a precursor or metabolic intermediate of estradiol. DU: digital ulcer; ILD: interstitial lung disease; JAKi: Janus kinase inhibitor; MSK: musculoskeletal; SERD: selective estrogen receptor degrader; SERM: selective estrogen receptor modulator; TOF: Tofacitinib.

An additional 62 genetic variants were reported in the GWAS Catalog but did not meet the pre-specified adjusted *P*-value cutoff. These were mapped to 55 genes, and, of these, only two genes were targeted by currently available drugs: *MMP9*, which is associated with five drugs, and *LAMC2*, which is targeted by one drug (see Supplementary Data File).

The genetic variants identified in the GWAS Catalog were gathered from 15 GWAS studies (see Supplementary Table S2 and the Supplementary Data File for details). Most studies, 12 in total, focused on individuals of European ancestry. Other ancestries were less represented, with Asian ancestry included in four studies and African and Middle Eastern ancestries represented in one study each.

### Groupings of drug therapies for potential repurposing in SSc

**Figure 2:**
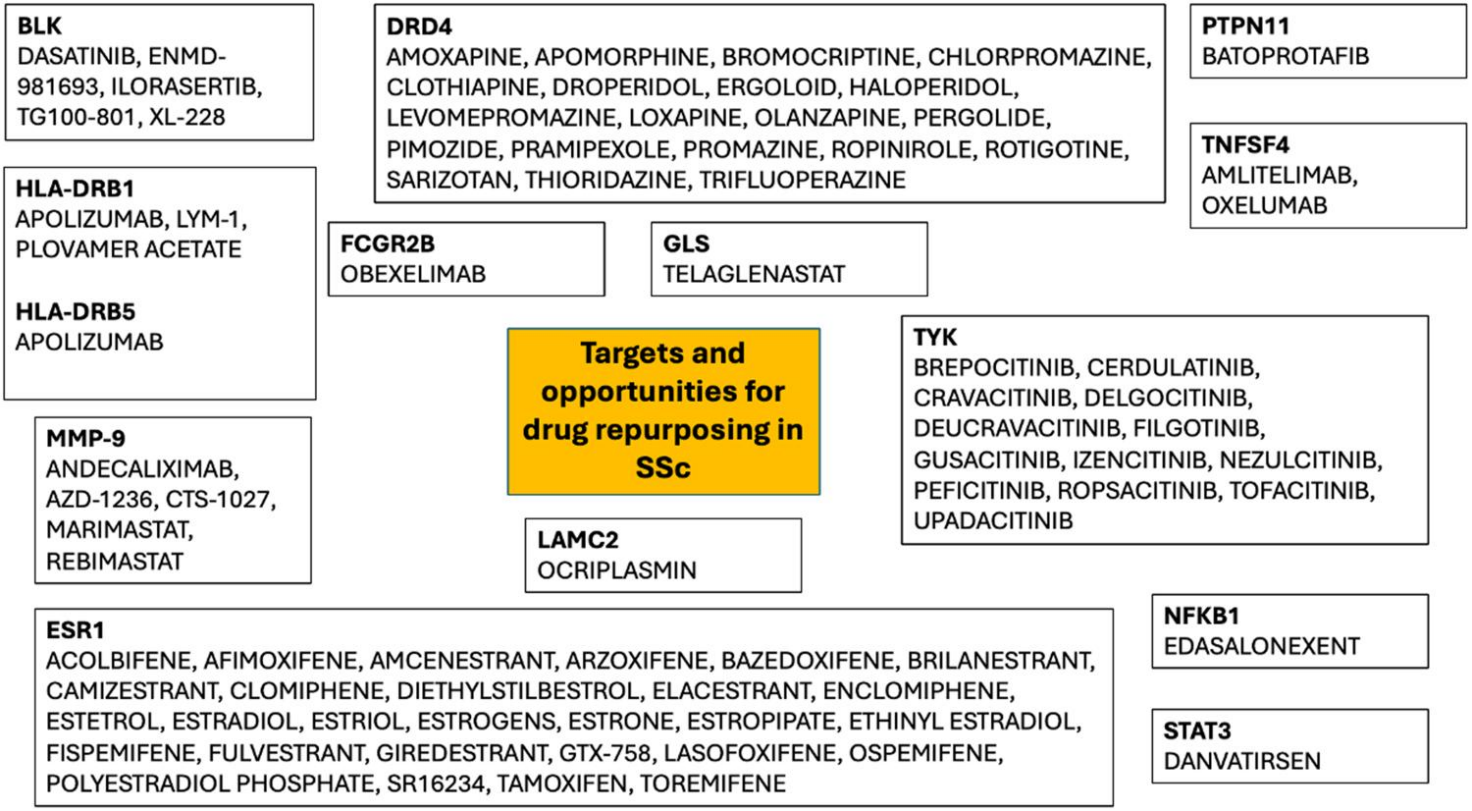
Drug targets and compounds identified for potential repurposing in SSc. ESR: estrogen receptor 1

Many drug therapies could conceptually be grouped by drug class and/or shared effector mechanism. An overview of the identified drugs (and associated gene targets) is presented in Fig. 2.

#### Female sex hormones and function

The most common drug therapies identified for drug repurposing in SSc were those broadly encompassing female sex hormones and function. These included selective estrogen receptor modulators (SERMs) (12 drugs) and selective estrogen receptor degraders (SERDs) (5 drugs). Other drugs were variations of oestrogen (including synthetic) hormone therapy: estradiol (E1), estrione (a precursor to E1) and esterol (E4). A selective estrogen receptor alpha agonist was also identified.

#### Neurotransmitter-targeting therapies

Neurotransmitter-targeting drug therapies, including those used for broad-ranging neurological disorders (e.g. movement disorders and psychosis), represented another apparent grouping. These included dopamine-targeting therapies (12 drugs) and drugs exerting effects through multiple neurotransmitter modulation, including dopamine/serotonin targeting therapies (4 drugs).

#### Inflammatory–fibrotic pathways

Immunosuppressive/modulatory approaches largely targeted the Janus Kinase (JAK)/STAT pathway: JAK inhibitors (JAKis; 7 drugs) and tyrosine kinase inhibitors (TYK) (4 drugs). We also identified dual JAK: TYK (1 drug) and spleen tyrosine kinase (SYK) (1 drug) inhibitors. Other therapies identified targeted anti-OX40L monoclonal antibody (two drugs) and protein kinase inhibitor (drugs) therapies, and a few other forms of immunosuppression/modulation of potential for repurposing in SSc. Concerning known complex matrix biological processes in the SSc, four MMPs were identified of potential interest. Danvatirsen is 16-nucleotide, generation 2.5 antisense oligonucleotide that down-regulates the expression of human *STAT3* mRNA and is being investigated as an anti-cancer therapy.

### Supporting evidence gained from existing studies in SSc

Overall, there are too few data to provide disease-specific evidence (safety and/or efficacy) for most of the identified drug therapies for potential repurposing specifically in the intended target population of patients with SSc, including evidence of completed and/or advanced clinical trial programs. Treatment with dasatinib (for 2 years) in a small open-label biomarker pilot study was reported to be associated with stability of radiographically assessed SSc-ILD in the majority of treated patients. Amlitelimab is currently being actively explored (NCT06195072) in the novel, placebo-controlled, Phase 2b platform CONQUEST RCT to investigate new treatments for patients with early SSc-ILD [27]. In a small pilot study, tofacitinib was associated with significant improvement in skin disease and with improvement in musculoskeletal involvement, and potential modification of digital ulcerative disease [28].

### Potential insights for SSc from broader treated patient populations

We took the opportunity to gain broader insights (safety and/or efficacy) from the reported experience of the identified drug therapies in other treated patient populations. We searched the extant literature for data of relevance to SSc, and to identify the latest stage of investigation reached in other diseases. The rationale for this being that later clinical trial programs would have had larger numbers of treated/exposed patients to therapies, including potentially post-marketing surveillance data. These data are presented in Supplementary Table S3.

A major concern of relevance to SSc is the safety ‘black box’ warning issued by authorities (e.g. the Food and Drug Administration and Medicines and Healthcare products Regulatory Agency) concerning JAKi treatment in patients with chronic inflammatory disorders [29]. Specifically, an increased incidence of major adverse cardiovascular events, malignancy, serious infections, venous thromboembolism and mortality, has been reported as a potential ‘class effect’ (and especially with tofacitinib) [29]. A few studies reported features of cold sensitivity (incorporating ‘classical’ RP) with treatment [apomorphine, arzoxifene, bazedoxifene, bromocriptine and deucravacitinib (nipple sensitivity)]; however, no definitive causality can be extrapolated from these treated study patient populations. Furthermore, an association with oestrogen replacement therapy and RP in post-menopausal women has been reported (compared with non-users), and this was more apparent in those treated with unopposed oestrogen, compared with combined oestrogen and progesterone [30].

## Discussion

We present the results of an integrated methodology to systematically identify potential drug targets for further evaluation and repurposing opportunities in SSc. We identified a wide range of possible candidates (*n* = 82) for drug repurposing in SSc. Many drug therapies could conceptually be grouped by drug class and/or shared effector mechanism/second: (i) female sex hormones and function, (ii) neurotransmitter-targeting therapies and (iii) inflammatory–fibrotic pathways. However, other approaches to grouping of drug therapies could be considered, for example, based on targeting disease manifestations (and recognizing that many drugs conceptually may confer benefit across multiple target domains).

Our approach ‘makes sense’ based on the known pathobiology of SSc, and identification of amlitelimab, provides proof of concept as this is being explored in the CONQUEST RCT for SSc-ILD. An unexpected and novel finding was the identification of many neurotransmitter-targeting therapies, thereby further justifying the approach taken in this study, which may have particular implications for Raynaud’s and gastrointestinal manifestations. However, it is important to highlight that these findings do not represent drugs that are immediately ready for repurposing, and that future work would be needed to validate their use in SSc.

Many drug therapies could conceptually be grouped by drug class and/or shared effector mechanism: (i) female sex hormones and function, (ii) neurotransmitter-targeting therapies and (iii) inflammatory–fibrotic pathways. Despite the striking female SSc predominance and apparent differences in phenotype/worse outcomes often observed in men, there are few data to explain these biological differences [31, 32]. However, it has been postulated (through the few translational studies of variable quality) that oestrogens may have possible profibrotic and vasodilatory effects in SSc [33]. Furthermore, an antifibrotic effect of raloxifene (a SERM) was reported in a disease model of SSc (generated using induced pluripotent stem cells–derived skin organoid) [34]. Aberrant activation of the JAK/STAT axis has been well described in biological samples of patients with SSc, likely mediated largely through IFN pathways [35], and has been linked with clinical expression of disease (including SSc-ILD) [36, 37]. Pre-clinical models support amelioration of fibrotic sequelae in SSc, and small studies in patients with SSc suggest significant potential clinical efficacy for skin involvement [36, 37]. A recent completed analysis of SSc patients enrolled in the multinational European Scleroderma Trials and Research Group cohort, provides support for generalized efficacy (skin, lung and musculocutaneous manifestations) of JAKis in SSc [38]. However, drug persistence and safety may be an important limiting factor in some patients with SSc, reflecting the recent ‘black box’ warnings issued by international authorities as a potential class effect for JAKis.

There was a broad repertoire of neurotransmitter-targeting therapies (including those used for neurology-based common conditions). Specifically, there is precedent for this in SSc, with growing evidence of dysautonomia impacting patients, evidence of enteric nervous system neuropathy [39] and evidence of sympathetic dysregulation in Raynaud’s; many of which occur early in the course of the disease [40]. Indeed, serotonin has been postulated to play an important link between vasculopathy and fibrosis in SSc [41], and the dopaminergic system has been implicated in autoimmune diseases, including key autocrine mediation through dendritic cells, and with amelioration of autoimmunity reported in animal models with dopamine agonists [42]. Furthermore, we identified danvatirsen (an antisense oligonucleotide targeting STAT3), which is of interest because broad epigenetic modifications have been reported in the complex pathobiology of SSc [3].

When developing drugs for SSc, it is crucial to consider off-target effects, as they can significantly limit clinical benefit and hinder the selection of appropriate drug targets. Prioritizing targets that are specific to the skin and/or involved organs, or employing targeted drug delivery to minimize off-target effects, could improve the risk–benefit profile.

Our study benefited from a pragmatic and integrated methodology, leveraging robust publicly available data; however, there are a few important aspects to highlight. The identified targets may have broader implications through related signalling pathways, or by further actions occurring upstream or downstream in the signalling pathway. Our current analysis does not account for this, as it did not screen for drugs acting on these related proteins. Expanding discovery efforts by incorporating pathway information, such as that found in Reactome [43], could reveal additional drug targets, though it may increase complexity. We acknowledge that potential novel pathways of mechanistic interest may have been missed by the approach. For example, a recent integrated genomic study using a machine learning approach identified novel risk loci and mechanistic pathways in SSc (especially highlighting *MICB*, *NOTCH4* and IFN-related genes) [44]. We specifically did not include these in our current analysis as these were not identified in the 15 examined GWAS; however, for completeness we found no repurposable drugs targeting for *MICB* and *NOTCH4* on a separate search of Open Targets (there is current interest in targeting the IFN-pathway in SSc).

Further limitations stem from inherent issues with evidence synthesis and data curation. There is some delay from the moment GWAS are published until the findings are made available within the GWAS Catalog and Ensembl. Furthermore, GWAS associations were treated equally, irrespective of the level of confirmed statistical significance. Future work could also further screen the identified original publications, informing the findings on the GWAS Catalog and Ensembl for data of interest from expression quantitative trait loci and colocalization, which could pinpoint further causal genes or drug targets of interest. Finally, the majority of the data come from studies involving individuals of European ancestry, and extrapolating and interpreting these findings in less represented ancestries must be done with caution.

Evolving knowledge on drug tractability and mechanisms of action, alongside additional genetic association discoveries, is likely to expand future drug development opportunities. Finally, a common theme is that many studies did not report outcomes relevant to patients with SSc (e.g. RP is almost universally observed) [45, 46], and this was usually more apparent for older studies (as might be expected).

In conclusion, we present the results from a robust methodology that has identified multiple possible candidates for drug repurposing in SSc. Our data provide biologic rationale and confirmation of disease effector mechanisms for current routes of clinical investigation. Future efforts could build on this work by elucidating the mechanisms underlying the identified targets, thereby helping to accelerate the typically lengthy and complex process of drug target identification and development. This may, in turn, open new avenues for exploring novel treatment approaches for SSc.

## Supplementary Material

keag084_Supplementary_Data

## Data Availability

Sharing of the data underlying this article will be considered on reasonable request to the corresponding author.
