# Supplementary material for: Advancing drug development for systemic sclerosis by prioritizing findings from human genetic association studies
Source: Rheumatology (Oxford). 2026 Feb 13;65(3):keag084. doi: 10.1093/rheumatology/keag084 (PMC13017111; doi:10.1093/rheumatology/keag084)
Supplement: keag084_Supplementary_Data [file keag084_supplementary_data.zip › rhe-25-2132-File002.docx]

**Supplementary material for ‘Advancing drug development for systemic sclerosis by prioritising findings from human genetic association studies’**

Michael Hughes PhD^1,2,3^, Zsuzsanna H McMahan MD^4^, Shervin Assassai MD^4^, Christopher P. Denton PhD^5^, Rui Providencia PhD^6,7^

Author affiliations:

1. Centre for Musculoskeletal Research, Division of Musculoskeletal and Dermatological Science, School of Biological Sciences, Faculty of Biological Medicine and Health, The University of Manchester, Manchester Academic Health Science Centre, Manchester, UK.
2. Department of Rheumatology, Northern Care Alliance NHS Foundation Trust, Salford Care Organisation, Salford, UK.
3. NIHR Manchester Biomedical Research Centre, Manchester University NHS Foundation Trust, Manchester, UK.
4. UTHealth Houston, Department of Medicine, Division of Rheumatology, Houston, Texas, USA.
5. Division of Medicine, University College London, London, UK.
6. Institute of Health Informatics, University College London, 222 Euston Road, NW1 2DA, UK.
7. Barts Heart Centre, St Bartholomew’s Hospital, West Smithfield, EC1A 7BE, UK.

| Risk Allele | P  Value | Mapped  Genes | locations | PubMedID | author | Discovery sample Ancestry (n) | Replication sample Ancestry (n) |
| --- | --- | --- | --- | --- | --- | --- | --- |
| HLA-DRB1*11:04-? | 2.00E-24 | - | - | 30247649 | González-Serna D | 764 Middle Eastern ancestry cases, 1,343 Middle Eastern ancestry controls | |
| rs11851053-C | 2.00E-11 | AHNAK2 | 14:104940871 | 38296975 | Ishikawa Y | 9,095 European ancestry cases, 17,584 European ancestry controls, 1,428 Japanese ancestry cases, 112,599 Japanese ancestry controls | |
| rs2819422-G | 3.00E-11 | AHNAK2 | 14:104942618 | 38296975 | Ishikawa Y | 1,428 Japanese ancestry cases, 112,599 Japanese ancestry controls | |
| rs4317244-G | 4.00E-08 | ANKRD37,UFSP2 | 4:185399752 | 33069728 | Pu W | 527 Han Chinese ancestry cases, 5,024 Han Chinese ancestry controls | 479 Han Chinese ancestry cases, 1,096 Han Chinese ancestry controls |
| rs1217393-? | 5.00E-09 | AP4B1-AS1 | 1:113891324 | 30573655 | Acosta-Herrera M | 2,281 European ancestry systemic sclerosis cases | |
| rs9884090-? | 2.00E-10 | ARHGAP31 | 3:119397303 | 31672989 | López-Isac E | 9,095 European ancestry cases, 17,584 European ancestry controls | |
| rs9884090-A | 6.00E-13 | ARHGAP31 | 3:119397303 | 38296975 | Ishikawa Y | 9,095 European ancestry cases, 17,584 European ancestry controls, 1,428 Japanese ancestry cases, 112,599 Japanese ancestry controls | |
| rs112846137-T | 1.00E-08 | ARL14,RPL6P8 | 3:160595133 | 30573655 | Acosta-Herrera M | 2,281 European ancestry systemic sclerosis cases European (Germany, Netherlands, Czech Republic, Hungary, Sweden, U.K., U.S., Italy, Spain) | |
| rs4134466-G | 7.00E-10 | ATG5 | 6:106129493 | 28314753 | Terao C | 564 Japanese ancestry cases, 1,863 Japanese ancestry controls, 2,592 European ancestry cases, 9,315 European ancestry controls | 564 Japanese ancestry cases, 1,863 Japanese ancestry controls, 2,592 European ancestry cases, 9,315 European ancestry controls |
| rs548234-C | 8.00E-11 | ATG5 | 6:106120159 | 38296975 | Ishikawa Y | 9,095 European ancestry cases, 17,584 European ancestry controls, 1,428 Japanese ancestry cases, 112,599 Japanese ancestry controls European (NR), East Asian (Japan) | |
| rs633724-T | 3.00E-09 | ATG5 | 6:106286165 | 31672989 | López-Isac E | 9,095 European ancestry cases, 17,584 European ancestry controls European (U.S., Australia, France, Germany, Netherlands, Norway, Sweden, U.K., Italy, Spain) | |
| rs633724-T | 2.00E-08 | ATG5 | 6:106286165 | 38296975 | Ishikawa Y | 9,095 European ancestry cases, 17,584 European ancestry controls, 1,428 Japanese ancestry cases, 112,599 Japanese ancestry controls | |
| rs802791-? | 4.00E-12 | ATG5 | 6:106121395 | 30573655 | Acosta-Herrera M | 2,281 European ancestry systemic sclerosis cases European (Germany, Netherlands, Czech Republic, Hungary, Sweden, U.K., U.S., Italy, Spain) | |
| rs9373839-G | 4.00E-08 | ATG5 | 6:106207742 | 24387989 | Mayes MD | 671 European ancestry ACA-positive cases, 347 European ancestry ATA-positive cases, 815 European ancestry cases, 3,466 European ancestry controls | 1,387 European ancestry ACA-positive cases, 896 European ancestry ATA-positive cases, 1,734 European ancestry cases, 5,935 European ancestry controls |
| rs2409781-? | 3.00E-08 | BLK | 8:11502048 | 29293537 | Gorlova OY | 1,833 European ancestry cases, 3,466 European ancestry controls, 291 African American cases, 260 African American controls | |
| rs2736346-A | 2.00E-12 | BLK | 8:11501603 | 30572963 | Márquez A | 3,477 European ancestry cases, 22,308 European ancestry controls | |
| rs2736340-? | 1.00E-08 | BLK,FAM167A | 8:11486464 | 23740937 | Martin JE | 2,761 European ancestry systemic sclerosis cases | 1,578 European ancestry systemic sclerosis cases |
| rs2736340-C | 1.00E-26 | BLK,FAM167A | 8:11486464 | 38296975 | Ishikawa Y | 9,095 European ancestry cases, 17,584 European ancestry controls, 1,428 Japanese ancestry cases, 112,599 Japanese ancestry controls | |
| rs2736340-T | 3.00E-21 | BLK,FAM167A | 8:11486464 | 31672989 | López-Isac E | 9,095 European ancestry cases, 17,584 European ancestry controls | |
| rs2056626-? | 3.00E-09 | CD247 | 1:167451188 | 20383147 | Radstake TR | 2,296 European ancestry cases, 5,171 European ancestry controls | 2,753 European ancestry cases, 4,569 European ancestry controls |
| rs2056626-? | 1.00E-11 | CD247 | 1:167451188 | 31672989 | López-Isac E | 9,095 European ancestry cases, 17,584 European ancestry controls | |
| rs2651804-? | 3.00E-10 | CD81-AS1,RNU6-878P | 11:2327389 | 31672989 | López-Isac E | 9,095 European ancestry cases, 17,584 European ancestry controls | |
| rs6598008-? | 2.00E-08 | CDHR5 | 11:618172 | 31672989 | López-Isac E | 9,095 European ancestry cases, 17,584 European ancestry controls | |
| rs685985-? | 4.00E-08 | CHD7,RAB2A | 8:60652405 | 31672989 | López-Isac E | 9,095 European ancestry cases, 17,584 European ancestry controls | |
| rs1378942-A | 9.00E-15 | CSK | 15:74785026 | 38296975 | Ishikawa Y | 9,095 European ancestry cases, 17,584 European ancestry controls, 1,428 Japanese ancestry cases, 112,599 Japanese ancestry controls | |
| rs1378942-C | 2.00E-14 | CSK | 15:74785026 | 31672989 | López-Isac E | 9,095 European ancestry cases, 17,584 European ancestry controls European (U.S., Australia, France, Germany, Netherlands, Norway, Sweden, U.K., Italy, Spain) | |
| rs17340351-? | 1.00E-08 | CYCSP20,TPI1P2 | 7:129068743 | 29293537 | Gorlova OY | 347 European ancestry cases, 3,466 European ancestry controls, 69 African American cases, 260 African American controls | |
| rs17340351-? | 2.00E-10 | CYCSP20,TPI1P2 | 7:129068743 | 29293537 | Gorlova OY | 1,833 European ancestry cases, 3,466 European ancestry controls, 291 African American cases, 260 African American controls | |
| rs17340646-G | 2.00E-08 | CYCSP20,TPI1P2 | 7:129082460 | 38296975 | Ishikawa Y | 9,095 European ancestry cases, 17,584 European ancestry controls, 1,428 Japanese ancestry cases, 112,599 Japanese ancestry controls | |
| rs10892286-C | 1.00E-10 | DDX6 | 11:118771376 | 38296975 | Ishikawa Y | 9,095 European ancestry cases, 17,584 European ancestry controls, 1,428 Japanese ancestry cases, 112,599 Japanese ancestry controls | |
| rs11217020-? | 2.00E-11 | DDX6 | 11:118768644 | 31672989 | López-Isac E | 9,095 European ancestry cases, 17,584 European ancestry controls | |
| rs11724804-A | 5.00E-11 | DGKQ | 4:971991 | 31672989 | López-Isac E | 9,095 European ancestry cases, 17,584 European ancestry controls | |
| rs11724804-A | 2.00E-10 | DGKQ | 4:971991 | 38296975 | Ishikawa Y | 9,095 European ancestry cases, 17,584 European ancestry controls, 1,428 Japanese ancestry cases, 112,599 Japanese ancestry controls | |
| rs13101828-G | 1.00E-08 | DGKQ | 4:971932 | 30573655 | Acosta-Herrera M | 2,281 European ancestry systemic sclerosis cases European (Germany, Netherlands, Czech Republic, Hungary, Sweden, U.K., U.S., Italy, Spain) | |
| rs35677470-A | 1.00E-20 | DNASE1L3 | 3:58197909 | 24387989 | Mayes MD | 1,127 European ancestry cases, 3,466 European ancestry controls | 2,507 European ancestry cases, 5,935 European ancestry controls |
| rs35677470-A | 3.00E-16 | DNASE1L3 | 3:58197909 | 24387989 | Mayes MD | 671 European ancestry ACA-positive cases, 347 European ancestry ATA-positive cases, 815 European ancestry cases, 3,466 European ancestry controls | 1,387 European ancestry ACA-positive cases, 896 European ancestry ATA-positive cases, 1,734 European ancestry cases, 5,935 European ancestry controls |
| rs35677470-A | 4.00E-31 | DNASE1L3 | 3:58197909 | 24387989 | Mayes MD | 671 European ancestry ACA-positive cases, 347 European ancestry ATA-positive cases, 815 European ancestry cases, 3,466 European ancestry controls | 1,387 European ancestry ACA-positive cases, 896 European ancestry ATA-positive cases, 1,734 European ancestry cases, 5,935 European ancestry controls |
| rs35677470-A | 1.00E-12 | DNASE1L3 | 3:58197909 | 30572963 | Márquez A | 3,477 European ancestry cases, 22,308 European ancestry controls | |
| rs35677470-A | 5.00E-09 | DNASE1L3 | 3:58197909 | 30573655 | Acosta-Herrera M | 2,281 European ancestry systemic sclerosis cases European (Germany, Netherlands, Czech Republic, Hungary, Sweden, U.K., U.S., Italy, Spain) | |
| rs193107685-C | 3.00E-09 | EIF4H,LIMK1 | 7:74123572 | 30573655 | Acosta-Herrera M | 2,281 European ancestry systemic sclerosis cases European (Germany, Netherlands, Czech Republic, Hungary, Sweden, U.K., U.S., Italy, Spain) | |
| rs10484921-A | 8.00E-09 | ESR1 | 6:151721125 | 38296975 | Ishikawa Y | 9,095 European ancestry cases, 17,584 European ancestry controls, 1,428 Japanese ancestry cases, 112,599 Japanese ancestry controls | |
| rs2736337-C | 5.00E-22 | FAM167A,BLK | 8:11484371 | 30573655 | Acosta-Herrera M | 2,281 European ancestry systemic sclerosis cases European (Germany, Netherlands, Czech Republic, Hungary, Sweden, U.K., U.S., Italy, Spain) | |
| rs4554699-? | 6.00E-14 | FCGR3B,FCGR2B | 1:161638578 | 29293537 | Gorlova OY | 671 European ancestry cases, 3,466 European ancestry controls, 21 African American cases, 260 African American controls | |
| rs4554699-? | 3.00E-13 | FCGR3B,FCGR2B | 1:161638578 | 29293537 | Gorlova OY | 347 European ancestry cases, 3,466 European ancestry controls, 69 African American cases, 260 African American controls | |
| rs4554699-? | 3.00E-08 | FCGR3B,FCGR2B | 1:161638578 | 29293537 | Gorlova OY | 1,833 European ancestry cases, 3,466 European ancestry controls, 291 African American cases, 260 African American controls | |
| rs76285340-T | 2.00E-11 | FLG2,CCDST | 1:152357053 | 33069728 | Pu W | 527 Han Chinese ancestry cases, 5,024 Han Chinese ancestry controls | 479 Han Chinese ancestry cases, 1,096 Han Chinese ancestry controls |
| rs7355798-T | 3.00E-10 | FLNB | 3:58145788 | 38296975 | Ishikawa Y | 9,095 European ancestry cases, 17,584 European ancestry controls, 1,428 Japanese ancestry cases, 112,599 Japanese ancestry controls | |
| rs3894194-A | 1.00E-10 | GSDMA | 17:39965740 | 28314753 | Terao C | 564 Japanese ancestry cases, 1,863 Japanese ancestry controls, 2,592 European ancestry cases, 9,315 European ancestry controls | 564 Japanese ancestry cases, 1,863 Japanese ancestry controls, 2,592 European ancestry cases, 9,315 European ancestry controls |
| rs883770-T | 5.00E-09 | GSDMB | 17:39907128 | 31672989 | López-Isac E | 9,095 European ancestry cases, 17,584 European ancestry controls | |
| rs883770-T | 1.00E-09 | GSDMB | 17:39907128 | 38296975 | Ishikawa Y | 9,095 European ancestry cases, 17,584 European ancestry controls, 1,428 Japanese ancestry cases, 112,599 Japanese ancestry controls | |
| rs2021408-C | 5.00E-84 | HLA-DPB1,HLA-DPA1 | 6:33078949 | 38296975 | Ishikawa Y | 463 Japanese ancestry cases, 112,599 Japanese ancestry controls | |
| rs2021408-C | 3.00E-57 | HLA-DPB1,HLA-DPA1 | 6:33078949 | 38296975 | Ishikawa Y | 575 Japanese ancestry cases, 112,599 Japanese ancestry controls | |
| rs2021408-C | 2.00E-62 | HLA-DPB1,HLA-DPA1 | 6:33078949 | 38296975 | Ishikawa Y | 625 Japanese ancestry cases, 112,599 Japanese ancestry controls | |
| rs987870-? | 2.00E-20 | HLA-DPB1,HLA-DPA1 | 6:33075103 | 21779181 | Gorlova O | 2,296 European ancestry cases, 5,172 European ancestry controls | 3,175 European ancestry cases, 4,210 European ancestry controls |
| rs3129882-? | 2.00E-27 | HLA-DRA | 6:32441753 | 21779181 | Gorlova O | 2,296 European ancestry cases, 5,172 European ancestry controls | 3,175 European ancestry cases, 4,210 European ancestry controls |
| rs3129763-? | 1.00E-11 | HLA-DRB1,HLA-DQA1 | 6:32623148 | 21779181 | Gorlova O | 2,296 European ancestry cases, 5,172 European ancestry controls | 3,175 European ancestry cases, 4,210 European ancestry controls |
| rs116526477-T | 3.00E-12 | HLA-DRB5,HLA-DRB9 | 6:32501102 | 38296975 | Ishikawa Y | 429 Japanese ancestry cases, 112,599 Japanese ancestry controls | |
| rs1767255821-AG | 4.00E-12 | HLA-DRB9,HLA-DRB5 | 6:32501499 | 38296975 | Ishikawa Y | 679 Japanese ancestry cases, 112,599 Japanese ancestry controls | |
| rs11177005-T | 2.00E-08 | IFNG-AS1,LINC01479 | 12:68015600 | 38296975 | Ishikawa Y | 429 Japanese ancestry cases, 112,599 Japanese ancestry controls | |
| rs45471499-A | 1.00E-09 | IGHM | 14:105855225 | 33069728 | Pu W | 527 Han Chinese ancestry cases, 5,024 Han Chinese ancestry controls | 479 Han Chinese ancestry cases, 1,096 Han Chinese ancestry controls |
| rs589446-? | 2.00E-10 | IL12A-AS1 | 3:160015740 | 31672989 | López-Isac E | 9,095 European ancestry cases, 17,584 European ancestry controls | |
| rs589446-T | 8.00E-10 | IL12A-AS1 | 3:160015740 | 38296975 | Ishikawa Y | 9,095 European ancestry cases, 17,584 European ancestry controls, 1,428 Japanese ancestry cases, 112,599 Japanese ancestry controls | |
| rs77583790-A | 2.00E-11 | IL12A-AS1 | 3:159976265 | 24387989 | Mayes MD | 1,127 European ancestry cases, 3,466 European ancestry controls | 2,507 European ancestry cases, 5,935 European ancestry controls |
| rs77583790-A | 1.00E-11 | IL12A-AS1 | 3:159976265 | 24387989 | Mayes MD | 671 European ancestry ACA-positive cases, 347 European ancestry ATA-positive cases, 815 European ancestry cases, 3,466 European ancestry controls | 1,387 European ancestry ACA-positive cases, 896 European ancestry ATA-positive cases, 1,734 European ancestry cases, 5,935 European ancestry controls |
| rs77583790-A | 2.00E-08 | IL12A-AS1 | 3:159976265 | 24387989 | Mayes MD | 671 European ancestry ACA-positive cases, 347 European ancestry ATA-positive cases, 815 European ancestry cases, 3,466 European ancestry controls | 1,387 European ancestry ACA-positive cases, 896 European ancestry ATA-positive cases, 1,734 European ancestry cases, 5,935 European ancestry controls |
| rs2305743-? | 5.00E-10 | IL12RB1 | 19:18082381 | 31672989 | López-Isac E | 9,095 European ancestry cases, 17,584 European ancestry controls | |
| rs2305743-A | 2.00E-08 | IL12RB1 | 19:18082381 | 38296975 | Ishikawa Y | 9,095 European ancestry cases, 17,584 European ancestry controls, 1,428 Japanese ancestry cases, 112,599 Japanese ancestry controls | |
| rs8109496-? | 4.00E-11 | IL12RB1 | 19:18079534 | 30572963 | Márquez A | 3,477 European ancestry cases, 22,308 European ancestry controls | |
| rs3790566-T | 4.00E-10 | IL12RB2 | 1:67348757 | 31672989 | López-Isac E | 9,095 European ancestry cases, 17,584 European ancestry controls European (U.S., Australia, France, Germany, Netherlands, Norway, Sweden, U.K., Italy, Spain) | |
| rs6659932-? | 6.00E-11 | IL12RB2 | 1:67336688 | 30573655 | Acosta-Herrera M | 2,281 European ancestry systemic sclerosis cases European (Germany, Netherlands, Czech Republic, Hungary, Sweden, U.K., U.S., Italy, Spain) | |
| rs6659932-A | 1.00E-11 | IL12RB2 | 1:67336688 | 32024964 | González-Serna D | 2,281 European ancestry systemic sclerosis cases, 1,988 European ancestry Crohn’s disease cases, 7,388 European ancestry controls | 3,453 European ancestry systemic sclerosis cases, 2,600 European ancestry Crohn’s disease cases, 7,180 European ancestry controls |
| rs2548998-G | 2.00E-11 | IRF1 | 5:132496822 | 32024964 | González-Serna D | 2,281 European ancestry systemic sclerosis cases, 1,988 European ancestry Crohn’s disease cases, 7,388 European ancestry controls | 3,453 European ancestry systemic sclerosis cases, 2,600 European ancestry Crohn’s disease cases, 7,180 European ancestry controls |
| rs10954214-T | 7.00E-17 | IRF5 | 7:128949579 | 30573655 | Acosta-Herrera M | 2,281 European ancestry systemic sclerosis cases European (Germany, Netherlands, Czech Republic, Hungary, Sweden, U.K., U.S., Italy, Spain) | |
| rs4728142-A | 2.00E-15 | IRF5 | 7:128933913 | 38296975 | Ishikawa Y | 9,095 European ancestry cases, 17,584 European ancestry controls, 1,428 Japanese ancestry cases, 112,599 Japanese ancestry controls | |
| rs10488631-? | 1.00E-09 | IRF5,TNPO3 | 7:128954129 | 29293537 | Gorlova OY | 574 European ancestry cases, 3,466 European ancestry controls, 201 African American cases, 260 African American controls | |
| rs10488631-? | 3.00E-10 | IRF5,TNPO3 | 7:128954129 | 29293537 | Gorlova OY | 1,087 European ancestry cases, 3,466 European ancestry controls, 82 African American cases, 260 African American controls | |
| rs10488631-? | 2.00E-10 | IRF5,TNPO3 | 7:128954129 | 21779181 | Gorlova O | 2,296 European ancestry cases, 5,172 European ancestry controls | 3,175 European ancestry cases, 4,210 European ancestry controls |
| rs10488631-? | 1.00E-09 | IRF5,TNPO3 | 7:128954129 | 21779181 | Gorlova O | 2,296 European ancestry cases, 5,172 European ancestry controls | 3,175 European ancestry cases, 4,210 European ancestry controls |
| rs10488631-C | 2.00E-13 | IRF5,TNPO3 | 7:128954129 | 20383147 | Radstake TR | 2,296 European ancestry cases, 5,171 European ancestry controls | 2,753 European ancestry cases, 4,569 European ancestry controls |
| rs11860650-? | 1.00E-10 | ITGAM | 16:31315385 | 23740937 | Martin JE | 2,761 European ancestry systemic sclerosis cases | 1,578 European ancestry systemic sclerosis cases |
| rs1635852-? | 2.00E-08 | JAZF1 | 7:28149792 | 23740937 | Martin JE | 2,761 European ancestry systemic sclerosis cases | 1,578 European ancestry systemic sclerosis cases |
| rs4731532-A | 2.00E-12 | KCP,IRF5 | 7:128932712 | 30572963 | Márquez A | 3,477 European ancestry cases, 22,308 European ancestry controls | |
| rs2275247-? | 1.00E-10 | KIAA0319L | 1:35442850 | 23740937 | Martin JE | 2,761 European ancestry systemic sclerosis cases | 1,578 European ancestry systemic sclerosis cases |
| rs11642873-? | 3.00E-10 | LINC01082,LINC02132 | 16:85958099 | 29293537 | Gorlova OY | 1,087 European ancestry cases, 3,466 European ancestry controls, 82 African American cases, 260 African American controls European (U.S., Spain), African American or Afro-Caribbean (U.S.) | |
| rs11642873-? | 2.00E-12 | LINC01082,LINC02132 | 16:85958099 | 21779181 | Gorlova O | 2,296 European ancestry cases, 5,172 European ancestry controls | 3,175 European ancestry cases, 4,210 European ancestry controls |
| rs12711490-? | 3.00E-10 | LINC01082,LINC02132 | 16:85939422 | 23740937 | Martin JE | 2,761 European ancestry systemic sclerosis cases | 1,578 European ancestry systemic sclerosis cases |
| rs35929052-? | 2.00E-09 | LINC01082,LINC02132 | 16:85960878 | 30573655 | Acosta-Herrera M | 2,281 European ancestry systemic sclerosis cases European (Germany, Netherlands, Czech Republic, Hungary, Sweden, U.K., U.S., Italy, Spain) | |
| rs2013112-T | 1.00E-09 | LINC01301 | 8:60484270 | 38296975 | Ishikawa Y | 9,095 European ancestry cases, 17,584 European ancestry controls, 1,428 Japanese ancestry cases, 112,599 Japanese ancestry controls | |
| rs398390-C | 2.00E-08 | LINC01967 | 3:28033182 | 38296975 | Ishikawa Y | 625 Japanese ancestry cases, 112,599 Japanese ancestry controls | |
| rs11117420-? | 4.00E-15 | LINC02132,LINC01082 | 16:85938316 | 31672989 | López-Isac E | 9,095 European ancestry cases, 17,584 European ancestry controls | |
| rs11117420-C | 1.00E-17 | LINC02132,LINC01082 | 16:85938316 | 38296975 | Ishikawa Y | 9,095 European ancestry cases, 17,584 European ancestry controls, 1,428 Japanese ancestry cases, 112,599 Japanese ancestry controls | |
| rs10499197-? | 2.00E-09 | LINC02539,WAKMAR2 | 6:137811379 | 23740937 | Martin JE | 2,761 European ancestry systemic sclerosis cases | 1,578 European ancestry systemic sclerosis cases |
| rs1450734198-TCTTAGCTATTGCTC | 3.00E-10 | LINC03072,RNU6-177P | 7:128575797 | 38296975 | Ishikawa Y | 1,428 Japanese ancestry cases, 112,599 Japanese ancestry controls | |
| rs2431098-G | 5.00E-12 | MIR3142HG | 5:160460329 | 30573655 | Acosta-Herrera M | 2,281 European ancestry systemic sclerosis cases European (Germany, Netherlands, Czech Republic, Hungary, Sweden, U.K., U.S., Italy, Spain) | |
| rs6457617-? | 4.00E-17 | MTCO3P1,HLA-DQB1 | 6:32696074 | 20383147 | Radstake TR | 2,296 European ancestry cases, 5,171 European ancestry controls | 2,753 European ancestry cases, 4,569 European ancestry controls |
| rs6457617-T | 2.00E-37 | MTCO3P1,HLA-DQB1 | 6:32696074 | 21750679 | Allanore Y | 564 European ancestry cases, 1,776 European ancestry controls | 1,682 European ancestry cases, 3,926 European ancestry controls |
| rs9275390-? | 3.00E-54 | MTCO3P1,HLA-DQB1 | 6:32701379 | 21779181 | Gorlova O | 2,296 European ancestry cases, 5,172 European ancestry controls | 3,175 European ancestry cases, 4,210 European ancestry controls |
| rs16832798-C | 5.00E-09 | NAB1 | 2:190669646 | 31672989 | López-Isac E | 9,095 European ancestry cases, 17,584 European ancestry controls European (U.S., Australia, France, Germany, Netherlands, Norway, Sweden, U.K., Italy, Spain) | |
| rs744600-? | 7.00E-11 | NAB1,GLS | 2:190700031 | 30573655 | Acosta-Herrera M | 2,281 European ancestry systemic sclerosis cases European (Germany, Netherlands, Czech Republic, Hungary, Sweden, U.K., U.S., Italy, Spain) | |
| rs57919238-A | 4.00E-08 | NBEA | 13:35459015 | 38296975 | Ishikawa Y | 625 Japanese ancestry cases, 112,599 Japanese ancestry controls | |
| rs17849502-T | 4.00E-15 | NCF2,SMG7 | 1:183563445 | 30573655 | Acosta-Herrera M | 2,281 European ancestry systemic sclerosis cases European (Germany, Netherlands, Czech Republic, Hungary, Sweden, U.K., U.S., Italy, Spain) | |
| rs230534-T | 5.00E-09 | NFKB1 | 4:102527884 | 31672989 | López-Isac E | 9,095 European ancestry cases, 17,584 European ancestry controls | |
| rs230534-T | 4.00E-12 | NFKB1 | 4:102527884 | 38296975 | Ishikawa Y | 9,095 European ancestry cases, 17,584 European ancestry controls, 1,428 Japanese ancestry cases, 112,599 Japanese ancestry controls | |
| rs443198-? | 9.00E-21 | NOTCH4 | 6:32222629 | 21779181 | Gorlova O | 2,296 European ancestry cases, 5,172 European ancestry controls | 3,175 European ancestry cases, 4,210 European ancestry controls |
| rs1005714-? | 2.00E-08 | NUP85 | 17:75228544 | 31672989 | López-Isac E | 9,095 European ancestry cases, 17,584 European ancestry controls | |
| rs1005714-G | 1.00E-08 | NUP85 | 17:75228544 | 38296975 | Ishikawa Y | 9,095 European ancestry cases, 17,584 European ancestry controls, 1,428 Japanese ancestry cases, 112,599 Japanese ancestry controls | |
| rs6679677-A | 2.00E-28 | PHTF1,RSBN1 | 1:113761186 | 30573655 | Acosta-Herrera M | 2,281 European ancestry systemic sclerosis cases European (Germany, Netherlands, Czech Republic, Hungary, Sweden, U.K., U.S., Italy, Spain) | |
| rs76246107-G | 3.00E-08 | PRR12 | 19:49618017 | 30573655 | Acosta-Herrera M | 2,281 European ancestry systemic sclerosis cases | |
| rs3130573-G | 6.00E-10 | PSORS1C2,PSORS1C1 | 6:31138491 | 21750679 | Allanore Y | 564 European ancestry cases, 1,776 European ancestry controls | 1,682 European ancestry cases, 3,926 European ancestry controls |
| rs11066301-T | 4.00E-08 | PTPN11 | 12:112433568 | 30573655 | Acosta-Herrera M | 2,281 European ancestry systemic sclerosis cases | |
| rs2476601-? | 2.00E-28 | PTPN22,AP4B1-AS1 | 1:113834946 | 30573655 | Acosta-Herrera M | 2,281 European ancestry systemic sclerosis cases European (Germany, Netherlands, Czech Republic, Hungary, Sweden, U.K., U.S., Italy, Spain) | |
| rs2176082-? | 5.00E-11 | PXK | 3:58345459 | 23740937 | Martin JE | 2,761 European ancestry systemic sclerosis cases | 1,578 European ancestry systemic sclerosis cases |
| rs4076852-A | 1.00E-08 | PXK | 3:58389559 | 38296975 | Ishikawa Y | 9,095 European ancestry cases, 17,584 European ancestry controls, 1,428 Japanese ancestry cases, 112,599 Japanese ancestry controls | |
| rs4076852-G | 1.00E-10 | PXK | 3:58389559 | 31672989 | López-Isac E | 9,095 European ancestry cases, 17,584 European ancestry controls European (U.S., Australia, France, Germany, Netherlands, Norway, Sweden, U.K., Italy, Spain) | |
| rs4681851-? | 4.00E-08 | PXK | 3:58410164 | 24387989 | Mayes MD | 671 European ancestry ACA-positive cases, 347 European ancestry ATA-positive cases, 815 European ancestry cases, 3,466 European ancestry controls | 1,387 European ancestry ACA-positive cases, 896 European ancestry ATA-positive cases, 1,734 European ancestry cases, 5,935 European ancestry controls |
| rs68191-C | 1.00E-10 | RN7SL26P,ZBTB9 | 6:33512961 | 32024964 | González-Serna D | 2,281 European ancestry systemic sclerosis cases, 1,988 European ancestry Crohn’s disease cases, 7,388 European ancestry controls | 3,453 European ancestry systemic sclerosis cases, 2,600 European ancestry Crohn’s disease cases, 7,180 European ancestry controls |
| rs2045526-G | 2.00E-10 | RNA5SP173,NDUFB5P1 | 4:179852838 | 38296975 | Ishikawa Y | 9,095 European ancestry cases, 17,584 European ancestry controls, 1,428 Japanese ancestry cases, 112,599 Japanese ancestry controls | |
| rs6697139-T | 5.00E-11 | RPL31P11 | 1:161690906 | 38296975 | Ishikawa Y | 1,428 Japanese ancestry cases, 112,599 Japanese ancestry controls | |
| rs7172677-? | 3.00E-08 | RPL36AP45,Metazoa_SRP | 15:75132252 | 23740937 | Martin JE | 2,761 European ancestry systemic sclerosis cases | 1,578 European ancestry systemic sclerosis cases |
| rs5862323-A | 3.00E-08 | RPS23P2,STMN1P2 | 4:137440228 | 38296975 | Ishikawa Y | 429 Japanese ancestry cases, 112,599 Japanese ancestry controls | |
| rs79834248-T | 3.00E-08 | RTEL1,RTEL1-TNFRSF6B | 20:63670906 | 38296975 | Ishikawa Y | 429 Japanese ancestry cases, 112,599 Japanese ancestry controls | |
| rs7929541-? | 2.00E-10 | SCT,DRD4 | 11:633689 | 30573655 | Acosta-Herrera M | 2,281 European ancestry systemic sclerosis cases | |
| rs9074-A | 2.00E-08 | SLC12A5 | 20:46060026 | 38296975 | Ishikawa Y | 9,095 European ancestry cases, 17,584 European ancestry controls, 1,428 Japanese ancestry cases, 112,599 Japanese ancestry controls | |
| rs4796791-T | 3.00E-08 | STAT3 | 17:42378745 | 32024964 | González-Serna D | 2,281 European ancestry systemic sclerosis cases, 1,988 European ancestry Crohn’s disease cases, 7,388 European ancestry controls | 3,453 European ancestry systemic sclerosis cases, 2,600 European ancestry Crohn’s disease cases, 7,180 European ancestry controls |
| rs10174238-? | 2.00E-09 | STAT4 | 2:191108308 | 29293537 | Gorlova OY | 671 European ancestry cases, 3,466 European ancestry controls, 21 African American cases, 260 African American controls | |
| rs10174238-? | 1.00E-11 | STAT4 | 2:191108308 | 29293537 | Gorlova OY | 1,087 European ancestry cases, 3,466 European ancestry controls, 82 African American cases, 260 African American controls | |
| rs10174238-? | 5.00E-13 | STAT4 | 2:191108308 | 29293537 | Gorlova OY | 1,833 European ancestry cases, 3,466 European ancestry controls, 291 African American cases, 260 African American controls | |
| rs10174238-? | 3.00E-42 | STAT4 | 2:191108308 | 30573655 | Acosta-Herrera M | 2,281 European ancestry systemic sclerosis cases | |
| rs11889341-T | 5.00E-12 | STAT4 | 2:191079016 | 38296975 | Ishikawa Y | 679 Japanese ancestry cases, 112,599 Japanese ancestry controls | |
| rs11889341-T | 3.00E-20 | STAT4 | 2:191079016 | 38296975 | Ishikawa Y | 1,428 Japanese ancestry cases, 112,599 Japanese ancestry controls | |
| rs11893432-? | 2.00E-08 | STAT4 | 2:191057148 | 29293537 | Gorlova OY | 1,087 European ancestry cases, 3,466 European ancestry controls, 82 African American cases, 260 African American controls | |
| rs11893432-? | 3.00E-12 | STAT4 | 2:191057148 | 24387989 | Mayes MD | 671 European ancestry ACA-positive cases, 347 European ancestry ATA-positive cases, 815 European ancestry cases, 3,466 European ancestry controls | 1,387 European ancestry ACA-positive cases, 896 European ancestry ATA-positive cases, 1,734 European ancestry cases, 5,935 European ancestry controls |
| rs11893432-? | 4.00E-11 | STAT4 | 2:191057148 | 29293537 | Gorlova OY | 1,833 European ancestry cases, 3,466 European ancestry controls, 291 African American cases, 260 African American controls | |
| rs13389408-C | 3.00E-17 | STAT4 | 2:191068557 | 30573655 | Acosta-Herrera M | 2,281 European ancestry systemic sclerosis cases | |
| rs3821236-A | 3.00E-09 | STAT4 | 2:191038032 | 20383147 | Radstake TR | 2,296 European ancestry cases, 5,171 European ancestry controls | 2,753 European ancestry cases, 4,569 European ancestry controls |
| rs3821236-A | 2.00E-23 | STAT4 | 2:191038032 | 31672989 | López-Isac E | 9,095 European ancestry cases, 17,584 European ancestry controls | |
| rs4853458-A | 2.00E-35 | STAT4 | 2:191094763 | 38296975 | Ishikawa Y | 9,095 European ancestry cases, 17,584 European ancestry controls, 1,428 Japanese ancestry cases, 112,599 Japanese ancestry controls | |
| rs7574865-T | 2.00E-13 | STAT4 | 2:191099907 | 21750679 | Allanore Y | 564 European ancestry cases, 1,776 European ancestry controls | 1,682 European ancestry cases, 3,926 European ancestry controls |
| rs7574865-T | 5.00E-10 | STAT4 | 2:191099907 | 28314753 | Terao C | 564 Japanese ancestry cases, 1,863 Japanese ancestry controls, 2,592 European ancestry cases, 9,315 European ancestry controls | 564 Japanese ancestry cases, 1,863 Japanese ancestry controls, 2,592 European ancestry cases, 9,315 European ancestry controls |
| rs7574865-T | 4.00E-12 | STAT4 | 2:191099907 | 33069728 | Pu W | 527 Han Chinese ancestry cases, 5,024 Han Chinese ancestry controls | 479 Han Chinese ancestry cases, 1,096 Han Chinese ancestry controls |
| rs7574865-T | 2.00E-13 | STAT4 | 2:191099907 | 21750679 | Allanore Y | 564 European ancestry cases, 1,776 European ancestry controls | 1,682 European ancestry cases, 3,926 European ancestry controls |
| rs7574865-T | 5.00E-10 | STAT4 | 2:191099907 | 28314753 | Terao C | 564 Japanese ancestry cases, 1,863 Japanese ancestry controls, 2,592 European ancestry cases, 9,315 European ancestry controls | 564 Japanese ancestry cases, 1,863 Japanese ancestry controls, 2,592 European ancestry cases, 9,315 European ancestry controls |
| rs7582694-C | 4.00E-10 | STAT4 | 2:191105394 | 38296975 | Ishikawa Y | 429 Japanese ancestry cases, 112,599 Japanese ancestry controls | |
| rs7601754-? | 3.00E-11 | STAT4 | 2:191075725 | 23740937 | Martin JE | 2,761 European ancestry systemic sclerosis cases | 1,578 European ancestry systemic sclerosis cases |
| rs7601754-A | 1.00E-09 | STAT4 | 2:191075725 | 38296975 | Ishikawa Y | 9,095 European ancestry cases, 17,584 European ancestry controls, 1,428 Japanese ancestry cases, 112,599 Japanese ancestry controls | |
| rs5029937-T | 2.00E-13 | TNFAIP3 | 6:137874014 | 38296975 | Ishikawa Y | 9,095 European ancestry cases, 17,584 European ancestry controls, 1,428 Japanese ancestry cases, 112,599 Japanese ancestry controls | |
| rs5029949-G | 2.00E-10 | TNFAIP3 | 6:137876369 | 38296975 | Ishikawa Y | 1,428 Japanese ancestry cases, 112,599 Japanese ancestry controls | |
| rs58721818-T | 5.00E-23 | TNFAIP3,LINC02865 | 6:137922602 | 30573655 | Acosta-Herrera M | 2,281 European ancestry systemic sclerosis cases | |
| rs10912594-C | 6.00E-12 | TNFSF4 | 1:173349525 | 38296975 | Ishikawa Y | 9,095 European ancestry cases, 17,584 European ancestry controls, 1,428 Japanese ancestry cases, 112,599 Japanese ancestry controls | |
| rs1857066-? | 5.00E-09 | TNFSF4 | 1:173363490 | 31672989 | López-Isac E | 9,095 European ancestry cases, 17,584 European ancestry controls | |
| rs2422345-A | 3.00E-08 | TNFSF4 | 1:173368608 | 30573655 | Acosta-Herrera M | 2,281 European ancestry systemic sclerosis cases | |
| rs844663-C | 5.00E-12 | TNFSF4 | 1:173274442 | 38296975 | Ishikawa Y | 9,095 European ancestry cases, 17,584 European ancestry controls, 1,428 Japanese ancestry cases, 112,599 Japanese ancestry controls | |
| rs1422673-T | 1.00E-08 | TNIP1 | 5:151059427 | 30572963 | Márquez A | 3,477 European ancestry cases, 22,308 European ancestry controls | |
| rs2233287-A | 5.00E-09 | TNIP1 | 5:151060536 | 21750679 | Allanore Y | 564 European ancestry cases, 1,776 European ancestry controls | 1,682 European ancestry cases, 3,926 European ancestry controls |
| rs3792783-G | 2.00E-12 | TNIP1 | 5:151076171 | 31672989 | López-Isac E | 9,095 European ancestry cases, 17,584 European ancestry controls European (U.S., Australia, France, Germany, Netherlands, Norway, Sweden, U.K., Italy, Spain) | |
| rs3792783-G | 2.00E-12 | TNIP1 | 5:151076171 | 38296975 | Ishikawa Y | 9,095 European ancestry cases, 17,584 European ancestry controls, 1,428 Japanese ancestry cases, 112,599 Japanese ancestry controls | |
| rs4958880-A | 1.00E-11 | TNIP1 | 5:151058916 | 30573655 | Acosta-Herrera M | 2,281 European ancestry systemic sclerosis cases | |
| rs960709-? | 2.00E-08 | TNIP1 | 5:151081488 | 23740937 | Martin JE | 2,761 European ancestry systemic sclerosis cases | 1,578 European ancestry systemic sclerosis cases |
| rs12531711-? | 5.00E-10 | TNPO3 | 7:128977412 | 29293537 | Gorlova OY | 1,833 European ancestry cases, 3,466 European ancestry controls, 291 African American cases, 260 African American controls | |
| rs12534421-? | 7.00E-10 | TNPO3 | 7:128984019 | 29293537 | Gorlova OY | 1,833 European ancestry cases, 3,466 European ancestry controls, 291 African American cases, 260 African American controls | |
| rs13238352-T | 1.00E-38 | TNPO3 | 7:129007888 | 30573655 | Acosta-Herrera M | 2,281 European ancestry systemic sclerosis cases | |
| rs36073657-T | 3.00E-21 | TNPO3 | 7:129011468 | 31672989 | López-Isac E | 564 European ancestry cases, 1,776 European ancestry controls | |
| rs62478615-? | 1.00E-10 | TNPO3 | 7:129044262 | 24387989 | Mayes MD | 671 European ancestry ACA-positive cases, 347 European ancestry ATA-positive cases, 815 European ancestry cases, 3,466 European ancestry controls | 1,387 European ancestry ACA-positive cases, 896 European ancestry ATA-positive cases, 1,734 European ancestry cases, 5,935 European ancestry controls |
| rs13239597-? | 1.00E-29 | TPI1P2 | 7:129055929 | 23740937 | Martin JE | 2,761 European ancestry systemic sclerosis cases | 1,578 European ancestry systemic sclerosis cases |
| rs9296015-? | 1.00E-08 | TSBP1-AS1,NOTCH4 | 6:32251212 | 21779181 | Gorlova O | 2,296 European ancestry cases, 5,172 European ancestry controls | 3,175 European ancestry cases, 4,210 European ancestry controls |
| rs11085725-? | 3.00E-10 | TYK2 | 19:10351837 | 30573655 | Acosta-Herrera M | 2,281 European ancestry systemic sclerosis cases European (Germany, Netherlands, Czech Republic, Hungary, Sweden, U.K., U.S., Italy, Spain) | |
| rs10892299-? | 2.00E-08 | Y_RNA,CXCR5 | 11:118856134 | 30572963 | Márquez A | 3,477 European ancestry cases, 22,308 European ancestry controls | |
| rs5754467-G | 1.00E-13 | YDJC,CCDC116 | 22:21630805 | 30573655 | Acosta-Herrera M | 2,281 European ancestry systemic sclerosis cases | |
| rs11171747-? | 6.00E-08 | ZC3H10,ESYT1 | 12:56124624 | 21779181 | Gorlova O | 2,296 European ancestry cases, 5,172 European ancestry controls | 3,175 European ancestry cases, 4,210 European ancestry controls |

**Supplementary Table S1.** List of unique genes identified by the search.

| PUBMED ID | Author | Year | Discovery sample A\ncestry (n) | Discovery sample Ancestry (country) | Replication sample Ancestry (n) | Replication sample Ancestry (country) |
| --- | --- | --- | --- | --- | --- | --- |
| 20383147 | Radstake TR | 2010 | 2,296 European ancestry cases, 5,171 European ancestry controls | European (U.S., Netherlands, Germany, Spain) | 2,753 European ancestry cases, 4,569 European ancestry controls | European (Sweden, U.S., Italy, Netherlands, Belgium, Germany, U.K., Spain, Norway) |
| 21779181 | Gorlova O | 2011 | 2,296 European ancestry cases, 5,172 European ancestry controls | European (U.S., Netherlands, Germany, Spain) | 3,175 European ancestry cases, 4,210 European ancestry controls | European (Sweden, U.S., Italy, Netherlands, Belgium, Germany, U.K., Spain, Norway) |
| 21750679 | Allanore Y | 2011 | 564 European ancestry cases, 1,776 European ancestry controls | European (France) | 1,682 European ancestry cases, 3,926 European ancestry controls | European (Italy, Germany, France) |
| 23740937 | Martin JE | 2013 | 2,761 European ancestry systemic sclerosis cases | European (U.S., Netherlands, Germany, Spain) | 1,578 European ancestry systemic sclerosis cases | European (Italy, U.K., Spain) |
| 24387989 | Mayes MD | 2014 | 1,127 European ancestry cases, 3,466 European ancestry controls | European (U.S., Spain) | 2,507 European ancestry cases, 5,935 European ancestry controls | European (U.S., Germany, Canada, Netherlands, Sweden, U.K., Italy, Spain) |
| 28314753 | Terao C | 2017 | 564 Japanese ancestry cases, 1,863 Japanese ancestry controls, 2,592 European ancestry cases, 9,315 European ancestry controls | East Asian (Japan), European (Canada, U.S., France, Germany, Italy) | 564 Japanese ancestry cases, 1,863 Japanese ancestry controls, 2,592 European ancestry cases, 9,315 European ancestry controls | East Asian (Japan), European (Canada, U.S., France, Germany, Italy) |
| 29293537 | Gorlova OY | 2018 | 1,833 European ancestry cases, 3,466 European ancestry controls, 291 African American cases, 260 African American controls | EuropeEuropean (U.S., Spain), African American or Afro-Caribbean (U.S.)a | NA |  |
| 30572963 | Márquez A | 2018 | 3,477 European ancestry cases, 22,308 European ancestry controls | European (Italy, Netherlands, Spain, U.K., U.S.) | NA |  |
| 30247649 | González-Serna D | 2019 | 764 Middle Eastern ancestry cases, 1,343 Middle Eastern ancestry controls | Greater Middle Eastern (Middle Eastern, North African or Persian) (Iran (Islamic Republic of), Turkey) | NA |  |
| 30573655 | Acosta-Herrera M | 2019 | 2,281 European ancestry systemic sclerosis cases | European (Germany, Netherlands, Czech Republic, Hungary, Sweden, U.K., U.S., Italy, Spain) | NA |  |
| 31672989 | López-Isac E | 2019 | 9,095 European ancestry cases, 17,584 European ancestry controls | European (U.S., Australia, France, Germany, Netherlands, Norway, Sweden, U.K., Italy, Spain) | NA |  |
| 32024964 | González-Serna D | 2020 | 2,281 European ancestry systemic sclerosis cases, 1,988 European ancestry Crohn’s disease cases, 7,388 European ancestry controls | European (Germany, Netherlands, Spain, U.K., U.S.) | 3,453 European ancestry systemic sclerosis cases, 2,600 European ancestry Crohn’s disease cases, 7,180 European ancestry controls | European (Germany, Italy, Spain, U.S.) |
| 33069728 | Pu W | 2021 | 527 Han Chinese ancestry cases, 5,024 Han Chinese ancestry controls | East Asian (China) | 479 Han Chinese ancestry cases, 1,096 Han Chinese ancestry controls | East Asian (China) |
| 38296975 | Ishikawa Y | 2024 | 9,095 European ancestry cases, 17,584 European ancestry controls, 1,428 Japanese ancestry cases, 112,599 Japanese ancestry controls | European (NR), East Asian (Japan) | NA |  |
| 40465716 | Liu TY | 2025 | 213 Han Taiwanese ancestry cases, 210,768 Han Taiwanese ancestry controls | East Asian (Taiwan) | NA |  |

**Supplementary Table S2.** List of GWAS studies providing information on associated genetic variants.

| Drug  (phase) | Main clinical Indication | Clinical evidence regarding drug SSc outcomes | Maximum level of evidence | Entries on PubMed* | Entries on Clinicaltrials.gov* |
| --- | --- | --- | --- | --- | --- |
| Acolbifene (2/3) | Breast cancer | - | - | 52 | 3 |
| Afimoxifene (2/3) | Breast cancer | - | - | 1251 | 19 |
| Amcenestrant (2) | Breast cancer | - | - | 16 | 9 |
| Amlitelimab (2b/3) | Atopic dermatitis, others (alopecia areata, asthma, coeliac disease, hidradenitis suppurativa) | - | - | 16 | 17 |
| Amoxapine (2) | Depression, psychotic symptoms, other (cocaine dependence) | - | - | 499 | 2 |
| Andecaliximab (3) | GI cancer, others (cystic fibrosis, COPD, Fibrodysplasia Ossificans Progressiva, UC) | - | - | 21 | 15 |
| Apolizumab (1/2) | Haematological malignancy | - | - | 23 | 6 |
| Apomorphine (3) | PD, others (pain, traumatic brain injury) | ‘Feeling cold’: Dose-titration phase (n=449) 1.7% & Long-term safety phase (n=426) = 0.3% (1) | Controlled study | 12,586 | 74 |
| Arzoxifene (3) | Breast cancer, osteoporosis, others, (gynaecological cancer) | ‘Vasodilation’: Arzoxifene 20mg (45.7%), 40mg (26.1%) (2) | Controlled study | 117 | 12 |
| AZD-1236 (2) | COPD | - | - | 0 | 6 |
| Batoprotafib (1) | Cancer | - | - | 0 | 0 |
| Bazedoxifene (3) | Postmenopausal osteoporosis, other (MS) | ‘Vasodilation’: placebo (n=1885), Bazedoxifene 20mg (n=1886), 40mg (1872) = 6.3%, 12.6%, 13.0% (3) | Controlled study | 560 | 55 |
| Brepocitinib (2/3) | Alopecia, DM, psoriasis, UC, uveitis | - | - | 51 | 6 |
| Brilanestrant (2) | Breast cancer | - | - | 3 | 0 |
| Bromocriptine (3/4) | Parkinson's disease, hyperprolactinaemia Others (Alzheimer’s, ALS, diabetes mellitus, peripartum cardiomyopathy) | Development of RP (4,5) | Case report | 9540 | 60 |
| Camizestrant (2/3) | Breast cancer | - | - | 16 | 12 |
| Cerdulatinib (2) | Atopic dermatitis, haematological malignancies | - | - | 44 | 4 |
| Chlorpromazine (3/4) | Antipsychotic, Others (cancer, COVID-19, hiccups) | - | - | 21,921 | 61 |
| Clomiphene  (3/4) | Infertility, Others (acromegaly) | - | - | 7530 | 270 |
| Clothiapine | Atypical antipsychotic | - | - | 82 | 2 |
| Cravacitinib | - | - | - | 1 | 0 |
| CTS-1027 | - | - | - | 3 | 4 |
| Danvatirsen (2) | Cancer (lung, pancreatic) | - | - | 14 | 6 |
| Dasatinib (3) | Haematological malignancy, breast cancer | Phase 3 study follow-up (varying drug doses), 2 cases (out of 670) developed reported PAH (RHC not performed) (6) | Controlled study | 5337 | 433 |
| Delgocitinib (3) | Eczema | - | - | 114 | 16 |
| Deucravacitinib (3) | Psoriasis | Phase 2 study – 19/ 21 (90%) patients described nipple hypersensitivity, but none discontinued (not clear if this included RP) (7) | - | 267 | 93 |
| Diethylstilbestrol (3) | Cancer (breast, prostate) | - | - | 10,737 | 53 |
| Droperidol (2/3) | Anti-emetic, sedation | - | - | 2914 | 49 |
| Edasalonexent (3) | Duchenne muscular dystrophy | - | - | 8 | 8 |
| Elacestrant (3/4) | Breast cancer | - | - | 92 | 29 |
| Enclomiphene (2/3) | Male infertility, secondary hypogonadism | - | - | 89 | 270 |
| ENMD-981693 (2) | Cancer | - | - | 0 | 0 |
| Ergoloid (3) | Behavioural/cognitive disorders, migraine | - | - | 763 | 2 |
| Estetrol (3) | Dysmenorrhea, pelvic pain, oral contraception | - | - | 271 | 34 |
| Estradiol (3/4) | Menopause-related conditions | - | - | 141,968 | 2626 |
| Estriol (2) | Menopause-related conditions | - | - | 8606 | 61 |
| Estrogens (3) | Menopause-related conditions | Post-menopausal women: adjusted OR for RP was 2.5 (95% CI, 1.2 to 5.3) for unopposed estrogen and 0.9 (CI, 0.3 to 2.6) for estrogen + progesterone, compared to non-users (8) | Controlled study | 305,370 | 4838 |
| Estrone (2) | Menopausal symptoms | - | - | 16,500 | 210 |
| Estropipate (3) | Menopausal symptoms, osteoporosis | - | - | 74 | 210 |
| Ethinyl estradiol (3) | Menopausal symptoms | - | - | 13,068 | 696 |
| Filgotinib (3) | RA, IBD | - | - | 458 | 66 |
| Fispemifene (3) | Post-menopausal symptoms, Hypogonadism in men | - | - | 285 | 4 |
| Fulvestrant (3) | Breast cancer | - | - | 4174 | 559 |
| Giredestrant (2/3) | Breast cancer | - | - | 26 | 20 |
| GTX-758 (2) | Prostate cancer | - | - | 4 | 4 |
| Gusacitinib (2) | Eczema | - | - | 13 | 1 |
| Haloperidol (3) | Anti-psychotic, post-operative vomiting | - | - | 23,794 | 251 |
| Ilorasertib (1) | Haematological malignancies | - | - | 5 | 4 |
| Izencitinib (2) | UC | - | - | 2 | 0 |
| Lasofoxifene (3) | Breast cancer | - | - | 186 | 9 |
| Levomepromazine (2) | Anti-emetic | - | - | 1097 | 9 |
| Loxapine (3) | Agitation in schizophrenia or bipolar disorder | - | - | 884 | 38 |
| LYM-1 (1) | B- and T-cell haematological malignancies | - | - | 157 | 3 |
| Marmiastat (3) | Cancer (prostate and lung), others (asthma) | - | - | 344 | 4 |
| Nezulcitinib (2) | Covid-19 | - | - | 6 | 1 |
| Obexelimab (2) | IgG4-related disease, others (MS, SLE, warm autoimmune haemolytic anaemia) | - | - | 14 | 4 |
| Ocriplasmin (3) | Vitreomacular adhesion | - | - | 395 | 28 |
| Olanzapine (3) | Anti-psychotic, others (anti-emetic e.g., chemotherapy-related) | - | - | 11,396 | 584 |
| Ospemifene (3) | Menopause-related conditions | - | - | 285 | 11 |
| Oxelumab (2) | Asthma | - | - | 65 | 0 |
| Peficitinib (3) | RA | - | - | 136 | 34 |
| Pergolide (2) | PD | - | - | 1237 | 18 |
| Pimozide (2) | Neurological disorders | - | - | 2694 | 18 |
| Plovamer acetate (2) | MS | - | - | 391,653 | 3 |
| Polyestradiol phosphate (2/3) | Prostate cancer | - | - | 214 | 314 |
| Pramipexole (3) | PD, restless legs, others (bipolar disorder) | - | - | 1844 | 189 |
| Promazine | Psychomotor agitation | - | - | 1360 | 0 |
| Rebimastat (2) | Cancer | - | - | 20 | 4 |
| Ropinirole (4) | PD, restless legs, others (ALS) | - | - | 1089 | 92 |
| Ropsacitinib (2) | Hidradenitis suppurativa, psoriasis | - | - | 10 | 0 |
| Rotigotine (4) | PD, others (restless less) | - | - | 707 | 115 |
| Sarizotan (2) | PD | - | - | 197 | 5 |
| SR16234 (2) | Endometrial disease including cancer | - | - | 16 | 1 |
| Tamoxifen (3/4) | Breast cancer | - | - | 34,280 | 690 |
| Telaglenastat (1/2) | Cancer | - | - | 44 | 17 |
| TG100-801 (1) | Macular degeneration | - | - | 0 | 2 |
| Thioridazine (1/2) | Anti-psychotic, others (AML) | - | - | 3336 | 11 |
| Tofacitinib (4) | AS, IBD, RA, PsA | FDA ‘black box warning' for JAKi – increased risk of MACE, VTE & cancer | Post-marketing surveillance | 4018 | 310 |
| Toremifene (3) | Breast cancer | Phase 3 study: ‘Peripheral coldness’ (2/126) patients treated with toremifene (9) | Controlled study | 818 | 39 |
| Trifluoperazine (2) | Anti-psychotic, breast cancer | - | - | 5287 | 8 |
| Upadacitinib (4) | AS, IBD, RA, PsA | FDA ‘black box warning'/MHRA warning for JAKi – increased risk of MACE, VTE, cancer & death | Post-marketing surveillance | 1542 | 146 |
| XL-228 (1) | Cancer | - | - | 4 | 2 |

**Supplementary Table S3**: Broader Clinical evidence on the available drugs targeting the identified hits and that may constitute repurposing opportunities outside of SSc. ALS: amyopathic lateral sclerosis; AS: ankylosing spondylitis; COPD: chronic obstructive pulmonary disease; DM: dermatomyositis; IBD: inflammatory bowel disease; JAKi: Janus kinase inhibitor; MACE: major adverse cardiovascular event; MS: multiple sclerosis; PD: Parkinson’s disease; PsA: psoriatic arthritis; RA: rheumatoid arthritis; UC: ulcerative colitis; VTE: venous thromboembolism.

**Supplementary Figure S1:** Brief Tutorial on Using the OpenTargets Platform

1. Loading the Platform


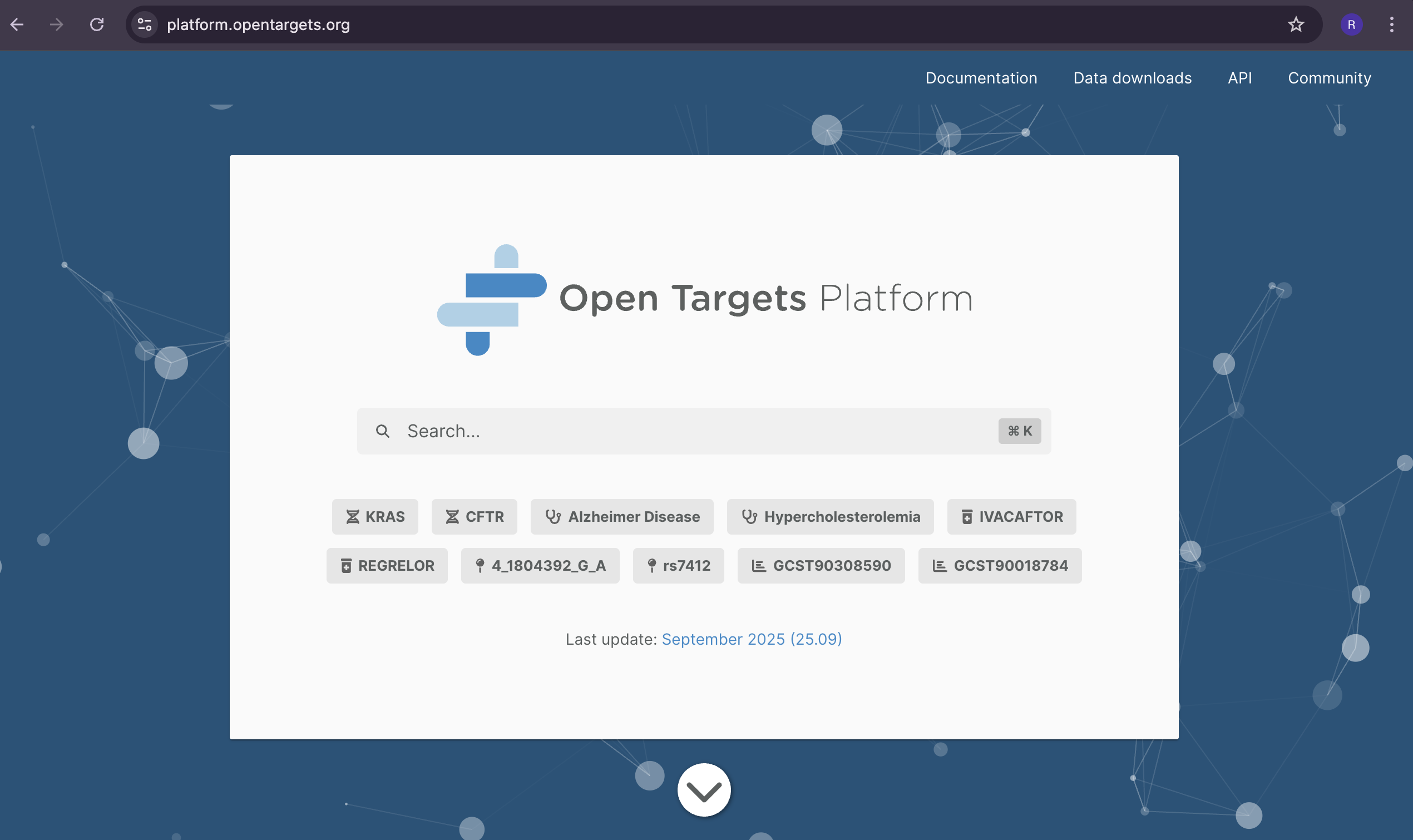


**B.** Typing the name of the gene of interest


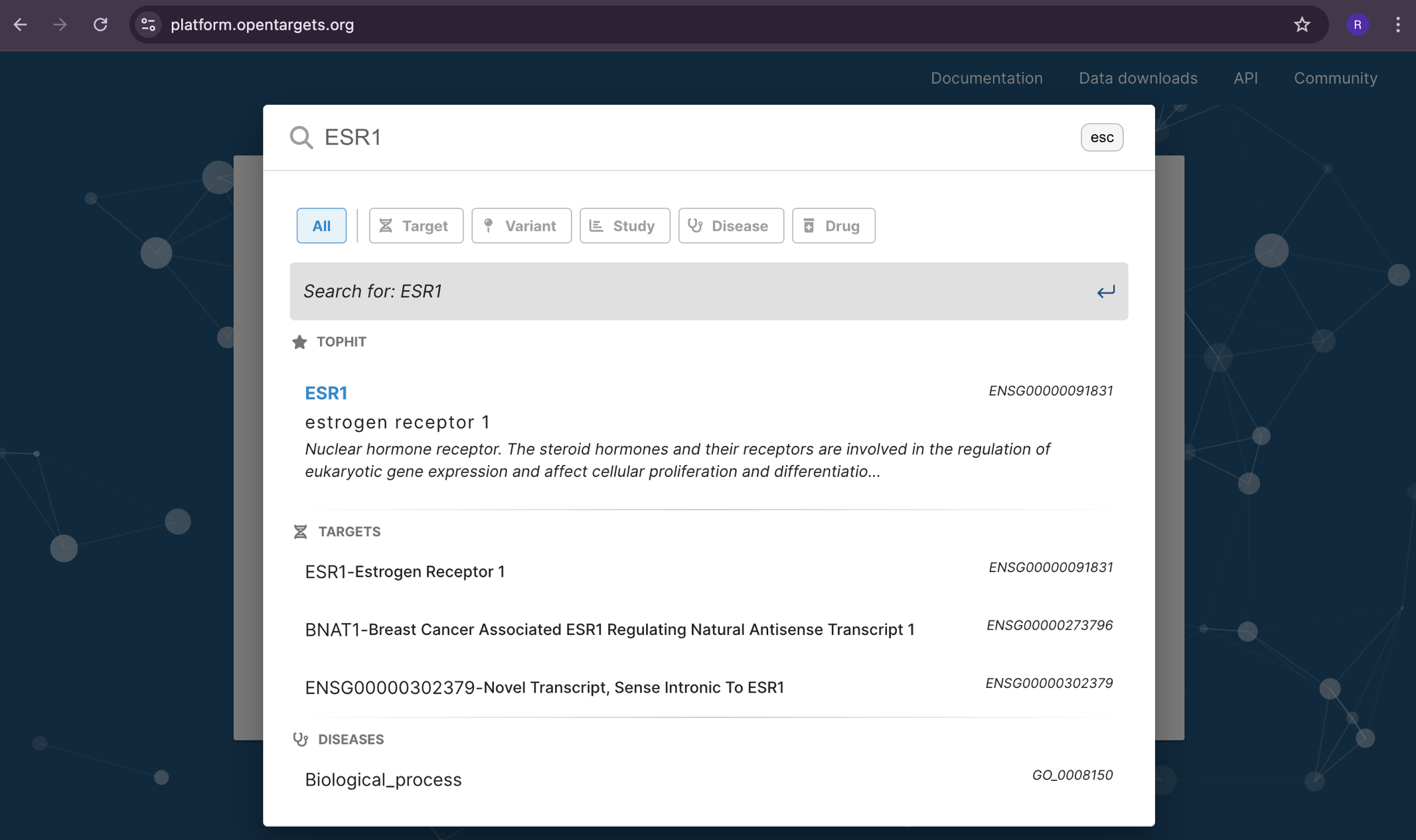


**C.** Accessing information on the gene of interest (identifiers and associated diseases)


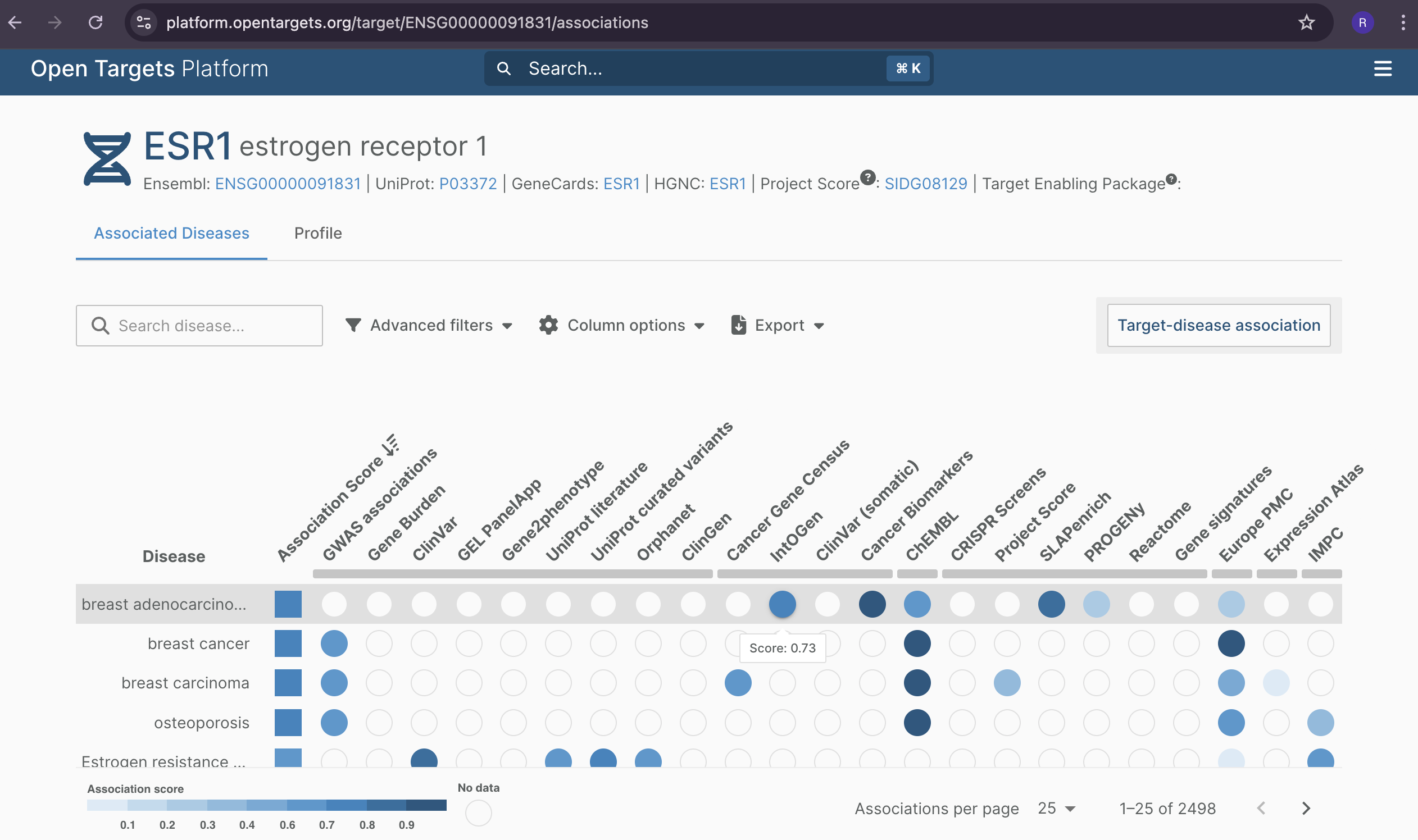


**D.** Changing tab for more information on the Profile of the gene


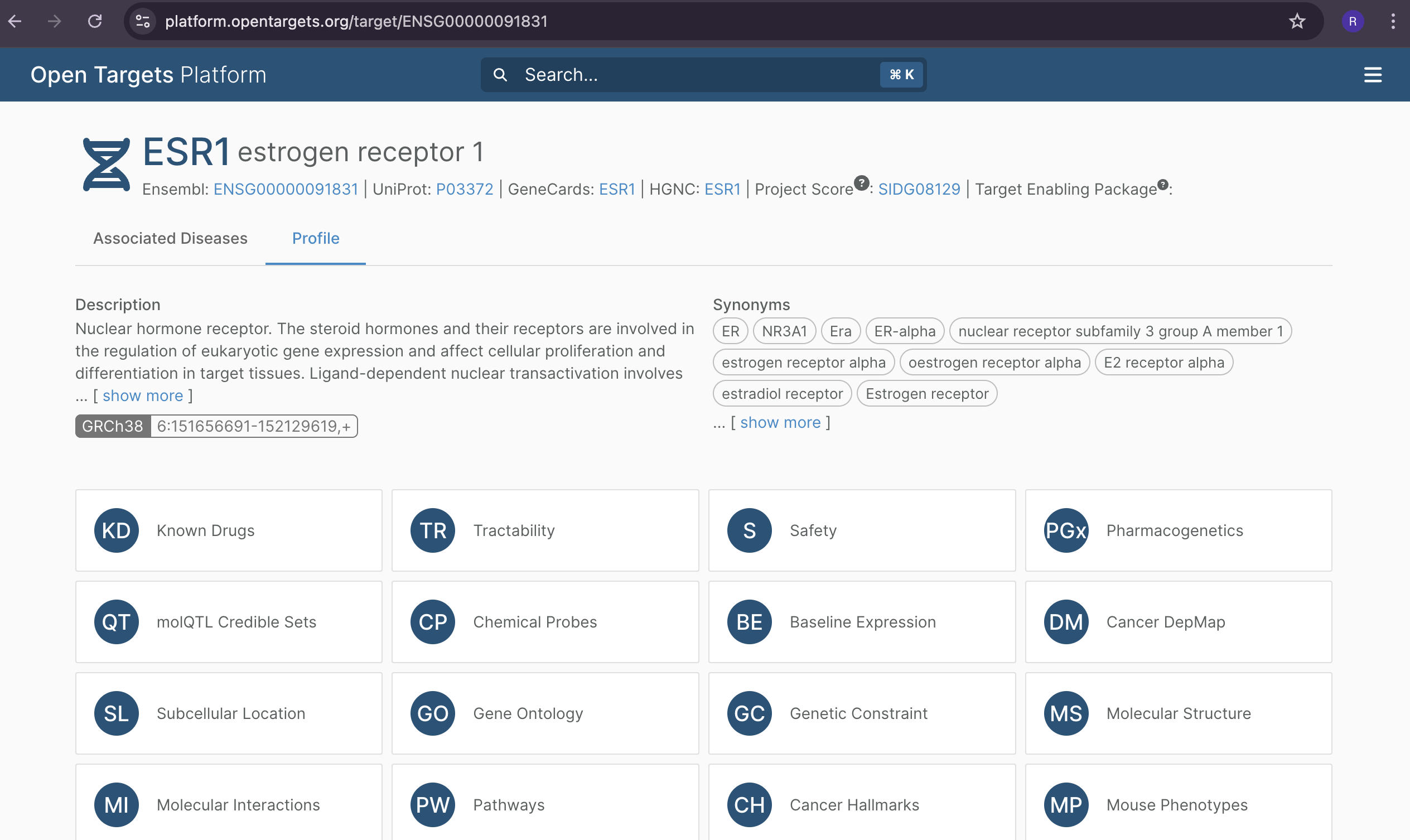


**E.** Detailed information on known drugs targeting the gene (can be exported)


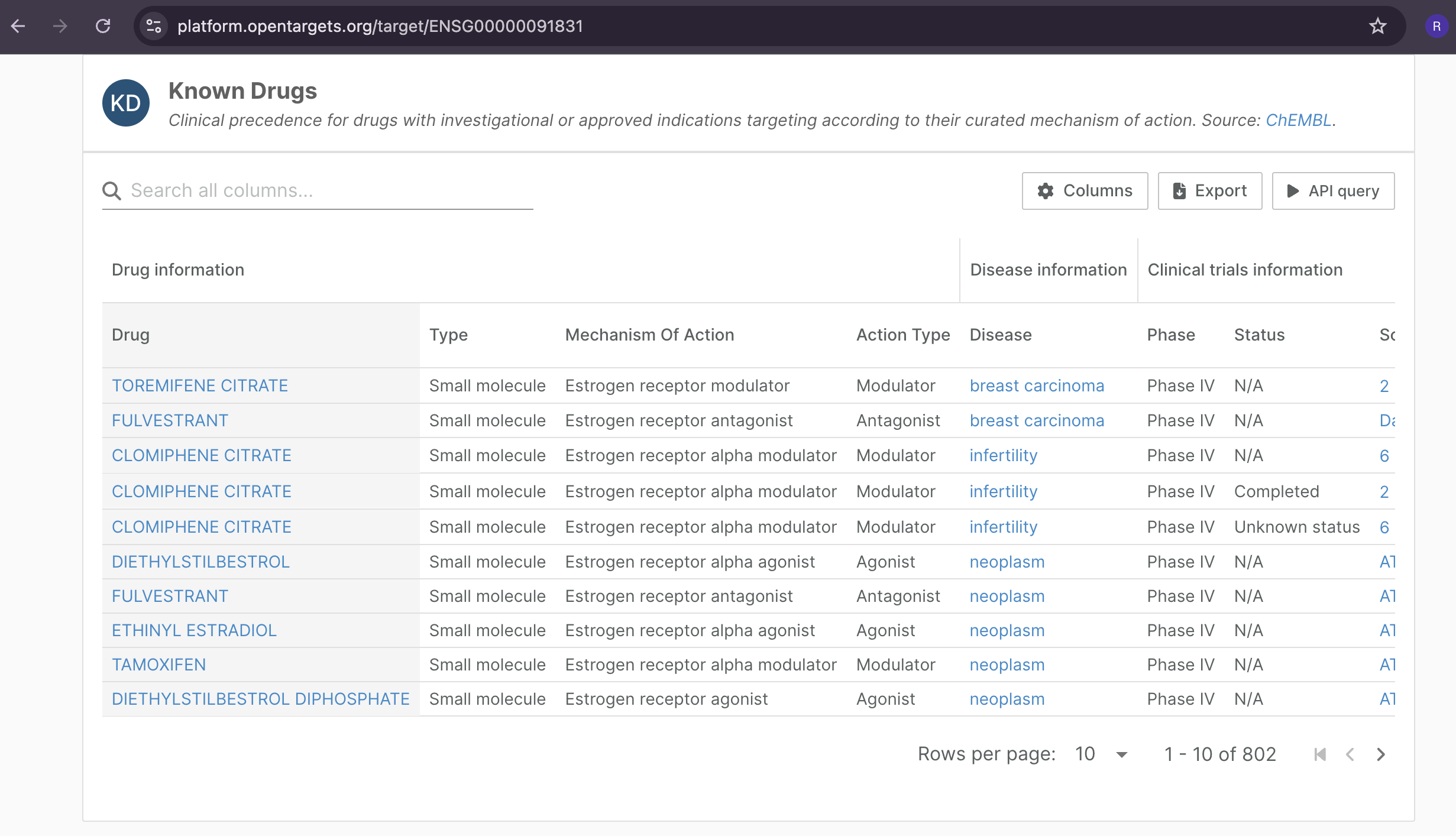


**F.** Information on Tractability & Gene Ontology


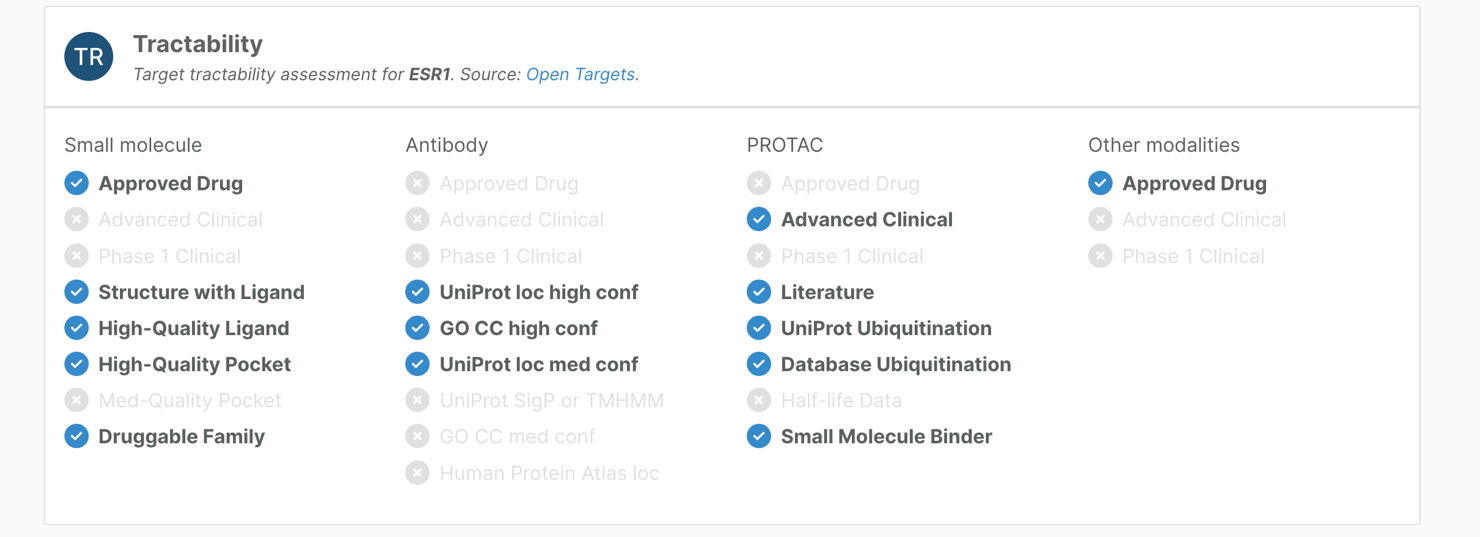


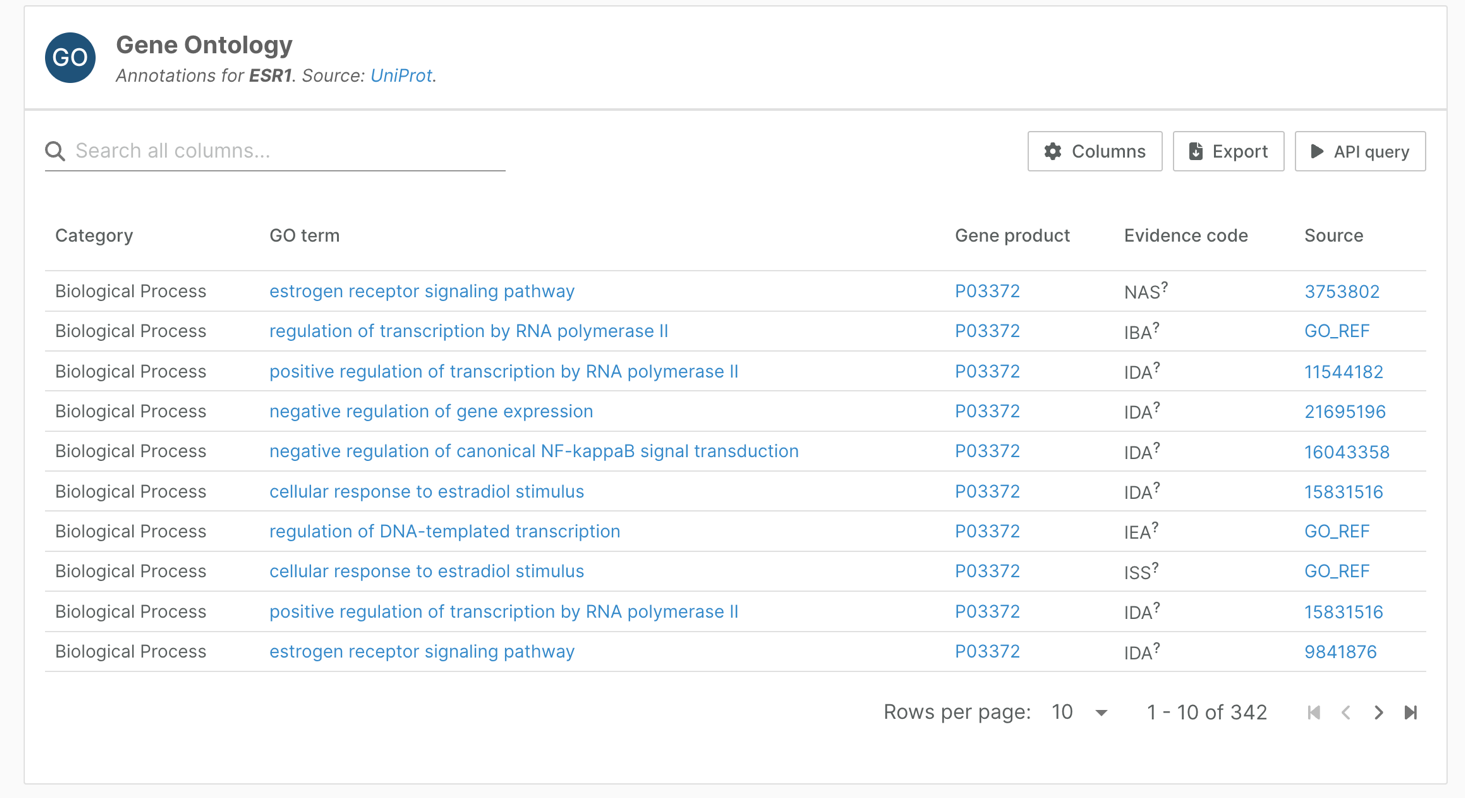


**References:**

1. Kassubek J, Factor SA, Balaguer E, Schwarz J, Chaudhuri KR, Isaacson SH, et al. Long-term safety, tolerability and efficacy of apomorphine sublingual film in  patients with Parkinson’s disease complicated by OFF episodes: a phase 3, open-label study. J Neurol 2024;271:3554–70.

2. Baselga J, Llombart-Cussac A, Bellet M, Guillem-Porta V, Enas N, Krejcy K, et al. Randomized, double-blind, multicenter trial comparing two doses of arzoxifene  (LY353381) in hormone-sensitive advanced or metastatic breast cancer patients. Ann Oncol 2003;14:1383–90.

3. Silverman SL, Christiansen C, Genant HK, Vukicevic S, Zanchetta JR, de Villiers TJ, et al. Efficacy of Bazedoxifene in Reducing New Vertebral Fracture Risk in Postmenopausal Women With Osteoporosis: Results From a 3-Year, Randomized, Placebo-, and Active-Controlled Clinical Trial. Journal of Bone and Mineral Research 2008;23:1923–34.

4. Khouri C, Blaise S, Carpentier P, Villier C, Cracowski JL, Roustit M. Drug-induced Raynaud’s phenomenon: beyond β-adrenoceptor blockers. Br J Clin Pharmacol 2016;82:6–16.

5. Quagliarello J, Barakat R. Raynaud’s phenomenon in infertile women treated with bromocriptine. Fertil Steril 1987;48:877–9.

6. Shah NP, Guilhot F, Cortes JE, Schiffer CA, le Coutre P, Brümmendorf TH, et al. Long-term outcome with dasatinib after imatinib failure in chronic-phase chronic  myeloid leukemia: follow-up of a phase 3 study. Blood 2014;123:2317–24.

7. Smith DC, Redman BG, Flaherty LE, LI L, Strawderman M, Pienta KJ. A phase II trial of oral diethylstilbesterol as a second-line hormonal agent in advanced prostate cancer. Urology 1998;52:257–60.

8. Fraenkel L, Zhang Y, Chaisson CE, Evans SR, Wilson PW, Felson DT. The association of estrogen replacement therapy and the Raynaud phenomenon in  postmenopausal women. Ann Intern Med 1998;129:208–11.

9. Kimura M, Tominaga T, Kimijima I, Takatsuka Y, Takashima S, Nomura Y, et al. Phase III randomized trial of toremifene versus tamoxifen for Japanese postmenopausal patients with early breast cancer. Breast Cancer 2014;21:275–83.
